# Sociohydrology: Scientific Challenges in Addressing the Sustainable Development Goals

**DOI:** 10.1029/2018WR023901

**Published:** 2019-08-16

**Authors:** Giuliano Di Baldassarre, Murugesu Sivapalan, Maria Rusca, Christophe Cudennec, Margaret Garcia, Heidi Kreibich, Megan Konar, Elena Mondino, Johanna Mård, Saket Pande, Matthew R. Sanderson, Fuqiang Tian, Alberto Viglione, Jing Wei, Yongping Wei, David J. Yu, Veena Srinivasan, Günter Blöschl

**Affiliations:** ^1^ Department of Earth Sciences Uppsala University Uppsala Sweden; ^2^ Centre of Natural Hazards and Disaster Science, CNDS Uppsala Sweden; ^3^ Department of Civil and Environmental Engineering University of Illinois at Urbana‐Champaign Urbana IL USA; ^4^ Department of Geography and Geographic Information Science University of Illinois at Urbana‐Champaign Urbana IL USA; ^5^ Agrocampus Ouest, INRA, UMR SAS Rennes France; ^6^ School of Sustainable Engineering and the Built Environment Arizona State University Tempe AZ USA; ^7^ GFZ German Research Centre for Geosciences Potsdam Germany; ^8^ Faculty of Civil Engineering and Geosciences Delft University of Technology Delft The Netherlands; ^9^ Department of Water Management Delft University of Technology The Netherlands; ^10^ Department of Hydraulic Engineering Tsinghua University Beijing China; ^11^ Institute of Hydraulic Engineering and Water Resources Management Vienna University of Technology Austria; ^12^ Department of Environment Land and Infrastructure Engineering (DIATI), Politecnico di Torino Turin Italy; ^13^ School of Earth and Environmental Sciences, University of Queensland Brisbane Queensland Australia; ^14^ Lyles School of Civil Engineering Purdue University West Lafayette IN USA; ^15^ Department of Political Science Purdue University West Lafayette IN USA; ^16^ Ashoka Trust for Research in Ecology and the Environment Bangalore India

**Keywords:** Sustainable Development Goals, water crises, sociohydrology, legacy effects, precautionary principle

## Abstract

The Sustainable Development Goals (SDGs) of the United Nations Agenda 2030 represent an ambitious blueprint to reduce inequalities globally and achieve a sustainable future for all mankind. Meeting the SDGs for water requires an integrated approach to managing and allocating water resources, by involving all actors and stakeholders, and considering how water resources link different sectors of society. To date, water management practice is dominated by technocratic, scenario‐based approaches that may work well in the short term but can result in unintended consequences in the long term due to limited accounting of dynamic feedbacks between the natural, technical, and social dimensions of human‐water systems. The discipline of sociohydrology has an important role to play in informing policy by developing a generalizable understanding of phenomena that arise from interactions between water and human systems. To explain these phenomena, sociohydrology must address several scientific challenges to strengthen the field and broaden its scope. These include engagement with social scientists to accommodate social heterogeneity, power relations, trust, cultural beliefs, and cognitive biases, which strongly influence the way in which people alter, and adapt to, changing hydrological regimes. It also requires development of new methods to formulate and test alternative hypotheses for the explanation of emergent phenomena generated by feedbacks between water and society. Advancing sociohydrology in these ways therefore represents a major contribution toward meeting the targets set by the SDGs, the societal grand challenge of our time.

## Key Points

- The crises that humanity faces over access to a clean water supply are increasingly connected and are growing in complexity
- Sociohydrology researchers must address several scientific challenges to strengthen basic knowledge and broaden the range of solvable problems
- Advances in sociohydrology research are progress toward meeting the targets defined by the United Nations' Sustainable Development Goals

## 1 Introduction: Water Crises, SDGs, and SocioHydrology

In the Anthropocene era, increasing attention is given in hydrologic science and water management to notions of *nonstationarity* (e.g., *stationarity is dead*; Milly et al., 2008) and *change* (e.g., *hydrology for a changing world*; Wagener et al., 2010). Yet, in the context of human influences on climate and hydrology, neither nonstationarity nor change is new. As early as 2,500 years ago, the pre‐Socratic Greek philosopher Heraclitus (circa 535–475 BCE) gained prominence for his emphasis on change as the fundamental essence of the universe. To express the concept that *nothing is permanent except change*, Heraclitus metaphorically referred to the change in the symbiotic relationship between water and people using the words *No man ever steps in the same river twice, for it's not the same river and he's not the same man*.

**Figure 1:**
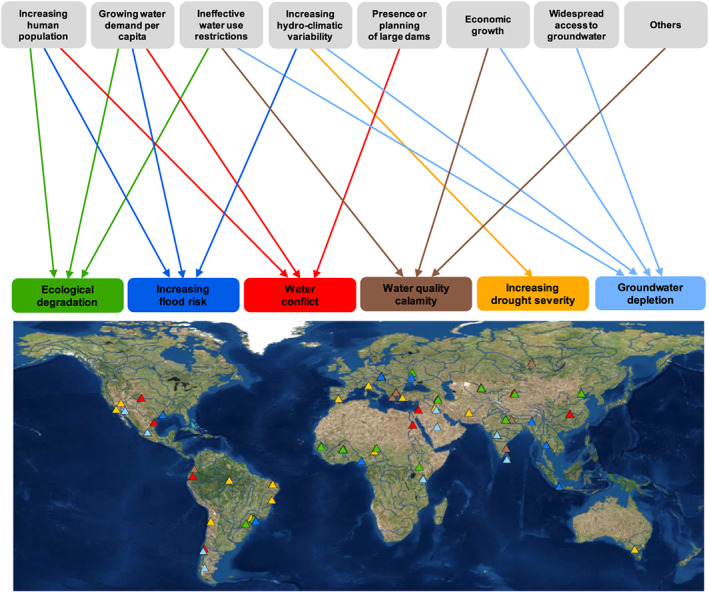
Global hot spots of water crises identified by the IAHS Panta Rhei survey across water scientists and experts. The figure shows the social, technical, and hydrological factors identified by the respondents as main drivers of the six types of water crises around the world. IAHS = International Association of Hydrological Sciences.

The prescient insight of Heraclitus can equally well serve as a metaphor for many of the water‐related issues humanity is currently facing worldwide. Millions of people around the world are affected by water crises manifesting at different scales, such as increasing drought severity and flood risk, groundwater depletion, ecological degradation, poor sanitation, water pollution, and its impact on human health (Srinivasan et al., 2012). A survey among water experts was recently carried out by the International Association of Hydrological Sciences (IAHS) to identify global hot spots of water crises (Figure 1). The results of the survey highlighted that most water crises are understood by experts as the result of lack of understanding, or neglect, of wider, economic, and socio‐cultural perspectives (by scientists, policy makers, and water resource managers). This supports the case that the global water crisis is a governance crisis and thus political, economic, and cultural in nature (Castro, 2007).

In the spirit of Heraclitus, therefore, the water crises can be deemed the intended and/or unintended consequences of long‐term changes (i.e., slow evolution) of social norms and values (or, more broadly, culture), ideology or political systems, which are not typically anticipated or accounted for in coping with water‐related issues. It is for this reason, and inspired by Heraclitus himself, that the global, decadal (2013–2022) initiative of the IAHS was titled Panta Rhei‐Everything Flows (McMillan et al., 2016; Montanari et al., 2013) and focuses on change in *both hydrology and society*.

Sociohydrology engages with these principles (Sivapalan et al., 2012, 2014), by examining the outcomes of water management and governance processes, that is, both *successes* and *failures*, themselves as subjects of scientific study. Sociohydrology studies the two‐way feedbacks between human and water systems that result in a wide range of *phenomena* that arise in different places around the world and in different contexts (Di Baldassarre et al., 2015; Gober & Wheater, 2015; Pande & Savenije, 2016; Sivapalan & Blöschl, 2015; Srinivasan, 2015; Srinivasan et al., 2012; Troy, Pavao‐Zuckerman, & Evans, 2015).

Work in sociohydrology has built upon a long history of work in three related fields. The first (1) is water resources systems (WRS) analysis that started with the Harvard Water Program in the 1960s (Brown et al., 2015; Kasprzyk et al., 2018) where the focus has mainly been on decision support by following a normative (optimization) route. The second (2) is integrated water resources management (IWRM), which was introduced in the 1990s and was more geared to actual implementation (Global Water Partnership, 2009) by (i) involving integration across the entire hydrological cycle; (ii) accommodating different water users and including engineering, economic, social, ecological, and legal aspects; while (iii) accounting for multiple spatial scales, such as upstream/downstream perspectives. The third (3) is the more recent development of interdisciplinary frameworks exploring the mutual shaping of society and nature (including water), such as social‐ecological systems, coupled human‐nature systems, and complex systems science (e.g., Adger, 2006; Cosens et al., 2018; Folke, 2006, 2010; Folke et al., 2010; Gohari et al., 2013; Liu et al., 2007; Loucks, 2015; Ostrom, 2009; Walker et al., 2004; Werner & McNamara, 2007).

While building on these fields, sociohydrology is different from them. WRS (1) analysis is focused on optimization. The goal is to combine hydrology and economics to design and operate *optimal infrastructure projects*. In contrast, the focus of sociohydrology is on *understanding why* certain water management outcomes arise rather than proposing actual management solutions. Similarly, IWRM (2) prescribes how to manage water resources in specific contexts, while sociohydrology analyzes actual water management processes and outcomes to develop generalizable understanding. Unlike social‐ecological systems and coupled human‐nature systems (3), sociohydrology has a more explicit focus on water, and on the specifics of the hydrologic cycle in space and time (Konar et al., 2018), including the role of water infrastructure (Di Baldassarre, Kemerink, et al., 2014; Di Baldassarre, Kreibich, et al., 2018). Over the past 6 years, sociohydrology has pursued understanding of several classes of emergent phenomena, which are actual outcomes, paradoxical dynamics, or unintended consequences that arise from water management in the context of human‐flood, human‐drought, and human‐environment interactions (Pande & Sivapalan, 2017; Sivapalan & Blöschl, 2015).

This paper argues that the development of generalized understanding of socio‐hydrological phenomena has an important role to play in informing policy processes and in assisting communities, governments, civil society organizations, and private actors as they mobilize to meet the United Nations Agenda 2030 and its Sustainable Development Goals, hereafter SDGs (United Nations, UN, 2015). The SDGs represent an ambitious blueprint to achieve a sustainable future for humanity, and address global challenges related to, among others, poverty, inequality, climate change, environmental degradation, and water (Death & Gabay, 2015; Fukuda‐Parr, 2016). Achieving the SDGs is urgent, and 193 nations have committed to meet the targets set by the United Nations by 2030. The SDG 6, *ensuring availability and sustainable management of water and sanitation for all* is probably the greatest challenge we face in water resources management (UN Water, 2018). However, water plays a key role in several of the other SDGs as well and, therefore, water management must account for these multiple interrelated objectives (either in synergy or in conflict), not just focusing on clean water and sanitation. SDG 6 (and other related SDGs) is strongly committed to the IWRM paradigm (UN Water, 2016). This, it is argued, requires governments *to consider how water resources link different parts of society and how decisions in one sector may affect water users in other sectors*, as well as to adopt a participatory and inclusive approach by involving *all actors and stakeholders, from all levels, who use and potentially pollute water, so that it is managed equitably and sustainably*. (UN Water, 2018).

Meeting the UN SDGs is, however, not straightforward. The targets set by the different SDGs are interrelated (UN Water, 2018), and they are sometimes fuzzy, contradictory, or challenging to implement (Sultana, 2018). For example, efforts to achieve the targets for clean water and sanitation can have unintended consequences on food and energy security and can contribute to environmental degradation. In the backdrop of these challenges, much of the current water management practice is still grounded on a strong techno‐managerial culture, focused more on technocratic approaches than on addressing the socio‐political and cultural dimensions underlying water crises, and the uneven distribution of costs and benefits (Hussein et al., 2018; Weststrate et al., 2018). Also, IWRM typically uses a scenario‐based approach to account for human‐water interactions (Savenije & Van der Zaag, 2008), which can work in the short term but in the long term can result in unintended consequences of only partially accounting for the coevolutionary dynamics of coupled human‐water systems (Di Baldassarre, Kooy, et al., 2013; Di Baldassarre, Viglione, et al., 2013; Di Baldassarre et al., 2015; Gohari et al., 2013; Sivapalan et al., 2012, 2014; Srinivasan et al., 2012). We posit that the field of sociohydrology has the potential to bridge the gap between the broad SDGs and the more specific IWRM/WRS set of principles and methodologies by seeking to gain insights that are both generalizable and actionable. Sociohydrology has an important role to play by emphasizing the need to broaden the conversation concerning water‐related issues so that they are addressed (i) holistically and inclusively, considering broader, socio‐cultural, and socio‐political perspectives; and (ii) by considering both short‐term and long‐term consequences of shifts in water governance.

This paper surveys the scientific challenges faced by sociohydrology toward addressing the complex water management challenge identified above. We start by documenting and synthesizing socio‐hydrological phenomena explored to date and the generalized understanding gained so far. Next, we discuss and highlight the scientific and methodological challenges that remain, and the opportunities toward integrating the social and hydrological sciences. This is essential for strengthening the field of sociohydrology, and broadening its scope to underpin IWRM and support policy makers, governments, communities, and private sector organizations toward meeting the SDGs, which looms as a major societal grand challenge of the 21st century.

## 2 The Role of SocioHydrology in Conceptualizing the Water Implications of the SDGs

### 2.1 Classification of Phenomena

Humans have significantly influenced the hydrological regime (Falkenmark & Rockström, 2008; Vörösmarty et al., 2013), deliberately or not (Blöschl et al., 2013; Savenije et al., 2014). Meanwhile, hydrological change tends to shape human society (Di Baldassarre et al., 2017), which responds to water crises, droughts, and floods in multiple ways, formally or not (Adger et al., 2013). The bidirectional feedbacks between human and water systems generate patterns across places or even across different contexts, which are the *phenomena* of interest to sociohydrology. These phenomena are actual outcomes, paradoxical dynamics, or unintended consequences that arise from water management to achieve a desired societal objective. They can result from the prevailing of political, commercial, or financial interests that might exacerbate social inequalities and ineffectiveness in water management. In this sense, they might be considered as manifestations of mismatch of governance with the (time, space, or organizational) scale of the coupled human‐water system being governed (Cash et al., 2006) or of governance processes thick with politics (Castro, 2007). Such ignorance and mismatch can arise when governance is based on myopic, reductionist, or one‐size‐fits‐all thinking. Sociohydrology aims to understand the feedback mechanisms generating these phenomena and the power relations, trust, cultural beliefs, and cognitive biases, which strongly influence the way in which people alter, and adapt to, changing hydrological regimes. The ultimate goal is to prevent mismatches of governance, in the first place, or at least overcome their adverse effects.

**Figure 2:**
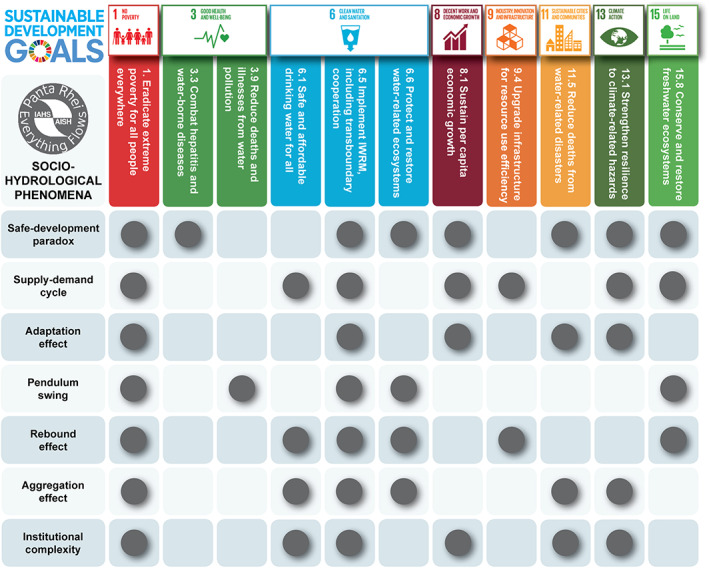
Water plays a key role in several specific targets of the SDGs, which are interconnected with socio‐hydrological phenomena. The SDGs thus provide further motivation and the necessity to broaden the scope and strengthen the foundation of sociohydrology, which requires integration of hydrological and social science perspectives. SDGs = Sustainable Development Goals.

Much of sociohydrology research has focused on the explanation of phenomena that have arisen in the context of floods (Di Baldassarre, Viglione, et al., 2013,Di Baldassarre, Kooy, et al., 2013, Di Baldassarre, Kemerink, et al., 2014,Di Baldassarre, Yan, et al., 2014; Di Baldassarre et al., 2015; Viglione et al., 2014; Grames et al., 2016; Ciullo et al., 2017; Barendrecht et al., 2019), droughts (Garcia et al., 2016; Srinivasan et al., 2017; Gonzales & Ajami, 2017; Di Baldassarre et al., 2017; Di Baldassarre, Kreibich, et al., 2018,Di Baldassarre, Wanders, et al., 2018; Treuer et al., 2017; Breyer et al., 2018), groundwater exploitation (Marston & Konar, 2017; Noël & Cai, 2017), water quality degradation (Chang et al., 2014; Giuliani et al., 2016), land degradation (Elshafei et al., 2014, 2016), farming and agriculture development (Fernald et al., 2015; Pande & Savenije, 2016), and water resources development (e.g., Chen et al., 2016; Kandasamy et al., 2014; Mostert, 2018; Srinivasan et al., 2012). Several studies have attributed the collapse or dispersal of ancient civilizations to unintended effects in water management or governance (e.g., Dermody et al., 2014; Kuil et al., 2016; Liu et al., 2013; Pande & Ertsen, 2014). Many of the phenomena studied to date manifest in the time domain. However, there is increased interest in phenomena that manifest in the space domain or in space‐time (Breyer et al., 2018; Chen et al., 2016), as also highlighted in the recent review by Konar et al. (2018). Much can be learned by comparing and contrasting phenomena that arise in different places and in different contexts, and by seeking common explanations. This can be done by organizing them into groups of similar behavior, as was earlier done in terms of *syndromes* by Srinivasan et al. (2012) in the context of water resource development. We next present several classes of socio‐hydrologic phenomena (Table 1) that have been explored over the last 6 years, providing the diversity of causes and contexts with which to generate understanding of coupled human‐water system dynamics and to help meet several water‐related SDG targets (see Figure 2).

**Table 1:** Overview of Socio‐Hydrological Phenomena and Implications of Understanding Socio‐Hydrological Phenomena for IWRM

General phenomenon	Main characteristics	Subphenomena	Implications for IWRM
Safe‐development paradox	Protection measures generate a false sense of security that reduces coping capacities thereby increasing social vulnerability.	*Levee effect*; White (1945)	•Focus on reducing social vulnerability
Kates et al. (2006)			•Better communication of water‐related risks
		*Reservoir effect*; Di Baldassarre, Wanders, et al. (2018)	
			•Proper quantification and pricing of risk by insurance companies
			•Enhanced integration of hard and soft path measures
Supply‐demand cycle	Increasing supply enables growth that in turn generates higher demands.	*Fixes that backfire*; Gohari et al. (2013)	•Focus on reducing demands rather than increasing supply
Kallis (2010)			
			•Price water accurately; scarcity value
			•Diversity water sources during drought; implement water conservation measures
Adaptation effect	Frequent extreme events increase coping capacities thereby reducing social vulnerability.	*Flood risk adaptation*; Kreibich et al. (2017)	•Focus on keeping adaptive capacities
Di Baldassarre et al. (2015)			
		*Sequence effect*; Di Baldassarre et al. (2017)	
			•Avoid maladaptive response to drought that might exacerbate future flood losses
	Adaptation to drought can worsen flood losses, and vice versa		
Pendulum swing	Changing priorities from pursuing economic prosperity or environmental protection	*Peak water paradoxes*; Gleick and Palaniappan (2010)	•Need to consider supply chain water use since local reduction in water use that accompany wealth may be offset by nonlocal water use increases
Kandasamy et al. (2014)		*Environmental Kuznets Curve*; Dinda (2004)	
Rebound effect	Increasing the efficiency leads to higher consumptions.	*Irrigation efficiency paradoxes*	•Implement governance for cap and trade system of water
Alcott (2005)		Dumont et al. (2013)	•Installing water efficient technologies is not necessarily going to lead to less water use.
			•Implement water basin use caps in addition to water efficient technologies
Aggregation effect	Undesirable outcomes at the system scale from aggregated optimal decisions at the individual scale	*Collective action*; Olson (1965) and Ostrom (1990)	•Implement systems level governance, for example, property rights for potential tragedy‐of‐the‐common cases
		*Water injustice*; Zwarteveen et al. (2017)	
			•Focus on the distribution of costs and benefits, not only average values
	Desirable outcomes at the system scale from aggregated inequalities at the individual scale		
			•Consider vulnerable communities
Institutional complexity	Trade‐off between resilience and efficiency	*Robustness‐fragility trade‐off*; Csete and Doyle (2002)	•Operationalize multi‐objective optimization, to, for example, make sure poor households do not get cutoff from water supply when pricing scheme is changed
			•Explicitly consider links between multiple systems

*Note*. IWRM = integrated water resources management.

According to the United Nations Office of Disaster Risk Reduction 2017 annual report (United Nations Office of Disaster Risk Reduction, UNISDR, 2018), capturing disaster risk dynamics is essential to achieve several SDGs. Shocks and stresses caused by disasters are likely to frustrate development achievements, and, in turn, *bad* development determines risk accumulation. Several SDGs are concerned with challenges that are related to risk accumulation, such as poverty (SDGs 1), reduction of inequalities (SDG 10), climate action (SDG 13), and peace, justice, and strong institutions (SDG 16). Sociohydrology has undertaken much work to understand feedbacks between society and hydrological extremes, such as droughts and flood. This work has advanced our understanding of the *safe‐development paradox* or levee effect. This phenomenon describes instances in which protection measures, such as levees, generate a false sense of security (Ludy & Kondolf, 2012) and build up social vulnerabilities in risky areas (Burton & Cutter, 2008). As a result, paradoxically, risk can even increase after building such structural protection measures (Di Baldassarre, Kreibich, et al., 2018). Considering this phenomenon is crucial to meet numerous SDGs (Figure 2). The levee effect, for example, shows that the target of reducing fatalities caused by water‐related disasters, such as floods (Target 11.5), cannot be achieved by merely building or reinforcing flood protection structures, but it requires a combination of nonstructural measures aiming to reduce vulnerabilities, including building codes or early warning systems (Kreibich et al., 2017).

The safe‐development paradox was first identified by Gilbert White as early as the 1940s (White, 1945), who criticized heavy reliance on structural flood protection in the United States. A recent example is the case of New Orleans, where a self‐reinforcing process of raising levees to protect a growing urban environment has taken place over many decades (Kates et al., 2006). This has led to extreme, low‐probability flooding, with catastrophic consequences, which are not evenly distributed across space and social groups. Masozera et al. (2007), for instance, analyzed the impact of flooding in New Orleans and found that preexisting socio‐economic conditions played a major role in the inability of particular social groups to respond to the disaster and to cope with the flooding.

Di Baldassarre, Wanders, et al. (2018) recently discussed the corresponding *reservoir effect* in the context of responses to droughts, showing how the safe‐development paradox can equally well apply to water supply reservoirs. These are often built to alleviate droughts and water shortages, but they can eventually worsen them. It is important to note that societies are not homogeneous and social stratification determines how and who are affected by hydrological extremes as well as who comes up with the strategies to cope with change at different scales, that is, how hydrological risks are distributed. This was recently demonstrated in the city of Austin, Texas, in the context of water restrictions imposed by the city in response to a severe drought (Breyer et al., 2018).

Dams, reservoirs, or other types of water infrastructure are often built to cope with drought and water scarcity. In the short‐term, these human alterations of water storage and fluxes are often beneficial, as they can increase water supply. Yet increasing water supply enables additional urban, agricultural, or economic growth that in turn generates higher demand, which can then offset the benefits of, for example, reservoirs as water supply sources. This phenomenon, known as *supply‐demand cycle* (Kallis, 2010), is a self‐reinforcing feedback, or vicious cycle, as the occurrence of a new drought will then likely favor further expansion of, for example, reservoirs to increase water supply (Di Baldassarre, Wanders, et al., 2018). Similar dynamics generated by water transfer projects in Iran have been described as *fixes that can backfire* (Gohari et al., 2013). It is important to acknowledge the role of social stratification and spatial distribution of water supply in terms of how and who makes decisions to build or expand water infrastructure, and who actually benefits from the increased water supply, as well as how the costs and benefits are distributed (Merme et al., 2014; Molle et al., 2009; Tiwale et al., 2018). This phenomenon has many implications for numerous SDGs (Figure 2), including the implementation of IWRM (Target 6.5).

Humans respond and adapt to hydrological extremes through a combination of spontaneous processes and deliberate strategies (Loucks et al., 2006, 2008) that can lead to, for example, changing the social contract (Adger et al., 2013). Adaptive responses can take place at the individual, community, or government level. They might lead to the emergence of the *adaptation effect*, that is, the negative impact of an extreme event tends to be lower if such an event occurs shortly after a similar one (Di Baldassarre et al., 2015; Kreibich et al., 2017). For instance, the economic losses of the 1995 Meuse River flooding in Central Europe were remarkably lower than those of 1993, even though the magnitudes of the two events were similar (Wind et al., 1999). Similarly, adaptation effects and decreasing flood fatalities have been observed in Bangladesh over the past decades (Kreibich et al., 2017; Mechler & Bouwer, 2015). While the adaptation effect is often associated with desirable outcomes, it can also have adverse consequences. Adaptation to drought conditions can exacerbate the negative impacts of floods, and vice versa (Di Baldassarre et al., 2017). For instance, changing reservoir operations to cope with drought, such as keeping the reservoirs as full as possible to buffer low flow conditions, can prevent required flood attenuation if heavy rainfall occurs during drought termination. This was seen, for example, during the catastrophic 2011 flooding in Brisbane that occurred shortly after the Millennium Drought in Australia or the extreme heavy rainfall causing the Oroville spillway collapse during the termination of the last multiyear drought in California (Mallakpour et al., 2019). Di Baldassarre et al. (2017) suggested that human migration from drought‐affected areas, as seen for example in Somalia (World Bank, 2018), can lead to more people living in riparian areas and therefore more exposed to flooding.

Many communities (be they agricultural or urban) that rely on river flow and/or groundwater to advance their economic livelihoods have been observed to swing between water extraction for food production or water control for urban development in the early stages, followed by efforts to mitigate and reverse degradation of the riparian environment, resulting from reduction of streamflows or depletion of groundwater. This is variously known as *pendulum swing*. This phenomenon was observed in the Murrumbidgee River basin in eastern Australia (Kandasamy et al., 2014), Lake Toolibin basin in Western Australia (Elshafei et al., 2014, 2016), and the Tarim basin in western China (Liu et al., 2015). In all three cases, increased water extraction, land clearance, and construction of water infrastructure are equally accompanied and driven by population growth and economic gain. In the short and intermediate terms, as per capita economic gain increases, the basin presents an attractive lifestyle proposition, causing human migration into the basin. In the long term, however, human actions that advance economic prosperity continue until the quantity or quality of water resources and the state of environment begin to impede further growth through the cost of environmental degradation and reduced productivity (Kandasamy et al., 2014). As the degradation of the environment continues, economic growth will naturally become constrained and communities will be compelled to act in efforts to reverse the negative threat to their livelihoods and well‐being.

In many arid regions of the world, water shortage most severely restricts socio‐economic development. Under such circumstances, developing highly efficient agriculture through water‐saving technology is regarded as an effective method to expand the economy, conserve water, and protect the environment. A range of technologies to increase irrigation efficiency and save water has proven successful at the farm scale. Yet, in the absence of appropriate governance measures, the increased efficiency often presents a paradox when assessed at larger scales because the saved water is often reallocated to irrigating an expanded land area or transferred to other uses (e.g., industrial or municipal water use), thus only to increase water consumption and deprive ecosystems of much needed flows. These are the unintended consequences arising from the push toward technological solutions without consideration of broader socio‐cultural behaviors and their consequences. This is known as the irrigation efficiency paradox and can be seen as a facet of an economic *rebound effect* or Jevons paradox (Alcott, 2005; Jevons, 1866). Increasing the efficiency of irrigation often leads to higher consumption of water, because farmers switch to more profitable and water‐consuming crops (Dumont et al., 2013). An example is the coupled use of water and energy in Mexico, where efficient and subsidized electricity supplied to pump groundwater for irrigation had the unintended effect of increasing the pumping, thus speeding up aquifer depletion (Scott, 2011; Scott et al., 2013). Similarly, in the Xinjiang Uygur Autonomous Region in western China, total water consumption continued to increase even as irrigation efficiency dramatically improved through the application of water‐saving technology (e.g., plastic mulching). However, the securing of additional freshwater resources through increased efficiency only encouraged farmers to expand the land area brought under irrigation, negating much of the water savings obtained through mulching (Liu, 2016; Zhang et al., 2014). This phenomenon is connected to numerous SDGs (Figure 2). Its emergence clearly complicates, for example, the goal of upgrading infrastructure to increase resource use efficiency (Target 9.4).

Other socio‐hydrological phenomena include what we term here as the *aggregation effect* and *institutional complexity*. The former relates to a mismatch of outcomes at the aggregated level of decisions taken at the individual level. A key example is known as collective action problem in the social sciences, which refers to situations where all individuals would be better off by jointly acting toward a common goal but fail to do so because of their self‐interest (Olson, 1965). This paradox originates from the common‐pool resource nature of water resources and the public good nature of water infrastructure and the combination of individual rationality and difficulties associated with coordination of large groups (Kollock, 1998). If overlooked, this issue can lead to outcomes such as overuse of groundwater and under provision of water infrastructure. To cope with this issue, rules and norms that regulate individual behavior need to be endogenously developed by the community or imposed on it endogenously (Ostrom, 1990). However, whether and how such governance arrangements can be achieved is itself a major challenge. Ostrom herself suggested in recent work (see, for instance, Van Laerhoven & Ostrom, 2007) that furthering common property resource management requires dealing with the uncertainty and complexity of institutional processes. Concurrently, over the past two decades, critical institutionalism has highlighted how the dynamic relationship between individuals, society, and natural resources is shaped by power relations and constant renegotiations that fuse socially embedded norms with new arrangements through a process of bricolage (Cleaver & De Koning, 2015; Rusca et al., 2015). *Getting institutions right* and development by design are, therefore, likely to lead to unintended outcomes (Cleaver, 2017). Feedbacks among the states of water resources, individual behavior, and change in governance arrangements can generate dynamics that are difficult to understand by treating water and human systems separately. Socio‐hydrological studies dealing with this phenomenon include the management of water infrastructure in relation to flooding (Yu, Sangwan, et al., 2017), irrigation (Muneepeerakul & Anderies, 2017; Yu et al., 2015), and groundwater exploitation (Madani & Dinar, 2012; Müller et al., 2017).

Aggregation effects could also produce perverse outcomes. These, for instance, occur when *desirable* outcomes at a larger scale conceal inequalities and, as such, distributional injustices at the local scale (Zwarteveen et al., 2017). A study on drinking water by Tiwale et al. (2018) found that additional reservoirs built to supply water for the underserved or unserved population of Lilongwe (Malawi) ended up improving continuity of supply for those who were already served, rather than quenching the thirst of the growing urban population. While at the urban scale water availability had improved, inequalities in access between different neighborhoods had increased. This example illustrates the limits of focusing on average values, while overlooking distribution across space and social groups. To determine whether anyone is *left behind*, as called for by the SDGs, requires disaggregated data, spanning from socio‐economic status, gender, age, and geographic location (Satterthwaite, 2016a, 2016b; Stuart & Woodroffe, 2016). As further elaborated below, sociohydrology can contribute to address important questions of equity by examining how development of large water infrastructure, such as reservoirs, and access to *extra* water intersects with gender, race, and socio‐economic status. These effects are also central to the three paradoxes identified in the most recent World Water Development Report (UN Water, 2019): (1) supplying the bulk of food and yet poor and hungry; (2) substantive investments in water infrastructure in rural areas and yet the rural poor lack access to water; and (3) small‐holder farmers being water productive and yet overlooked.

The *institutional complexity effect* relates to the trade‐off between resilience and efficiency of human‐water systems (Muneepeerakul & Anderies, 2017) generated by increased complexities of shared water infrastructure and related governance arrangements. Measures that increase performance and stability are preferred by managers. As such, coupled human‐water systems often evolve in ways that add more complex infrastructure and governance arrangements to reduce hydrological variability and increase system performance (Anderies, 2015). However, historical events showed that such complexities are often associated with hidden vulnerabilities to other types of disturbances, which are revealed through catastrophic failures (ibid). This line of inquiry has mostly focused on endogenous growth in the Indus and Hohokam civilizations (Pande & Ertsen, 2014) and virtual water trade in the Roman Empire (Dermody et al., 2014). Complex systems literature refers to this phenomenon as robustness‐fragility trade‐off (Csete & Doyle, 2002).

Overall, the phenomena discussed above provide significant insights into human‐water interactions at different temporal and spatial scales. These phenomena have clear implications for SDGs and can inform policies and strategies to achieve water‐related targets (Figure 2).

### 2.2 Explanation of the Phenomena

One of the main goals of sociohydrology is to explore the feedback mechanisms that may have caused the emergent phenomena. The driving logic of scientific discovery in sociohydrology is *if these feedback mechanisms operate, here is the phenomenon it can produce* as well as *if this phenomenon happens, here are the feedback mechanisms that might explain it* (Elster, 2007).

A suite of different methods has been utilized in various studies to provide these explanations. One common approach adopted in the social sciences is the use of statistical analysis of empirical research data, obtained through surveys and interviews (Brown, 2007; Daniel et al., 2018; Sanderson et al., 2017), some of which is qualitative. In this context, a set of interesting methods has been developed that combine the strengths of qualitative and quantitative data (Driscoll et al., 2007; Jick, 1979). An example is the work by Leong (2018) to convert narratives of perceptions of floods in Assam, India, to quantitative forms, similar to water volumes and prices, using the so‐called Q methodology. A second approach to explain phenomena, especially in the absence of long time series of observations, which has gathered increased momentum, is *agent‐based modeling* (Du et al., 2017; Noël & Cai, 2017). These models operate by prescribing rules on how individuals and/or institutions (the agents) interact, obtained through field surveys and interviews of people in real places, and thus allow the heterogeneous individual (or group) behavior to be accommodated. They help to compute the interactions at the microlevel between agents and allow describing social behaviors at the macrolevel or to interpret observed behavior at these higher levels and attribute them to both microscale and macroscale factors (Gilbert, 2008; Gilbert & Terna, 2000). A third common approach, called *system dynamics modeling* (e.g., Di Baldassarre, Viglione, et al., 2013; Garcia et al., 2016; Gohari et al., 2013; Srinivasan, 2015) is adopted in the presence of long time series of natural or water system behavior (e.g., hydrology, water use, and ecology) and social system behavior (e.g., demographics, economics, industries, and technology). The approach is guided by a limited number of hypotheses about fundamental natural and social processes and their interactions driving the overall behavior of the system, as illustrated by a stylized model for an urban socio‐hydrological system of Chennai, India, developed by Srinivasan (2015). More commonly, though, these hypotheses are explicitly formalized (in mathematical terms) using a set of coupled differential equations. Di Baldassarre et al. (2015) argue that *the strength of this method is its transparency, flexibility, and ability to capture the dynamics emerging from interacting processes*.

**Figure 3:**
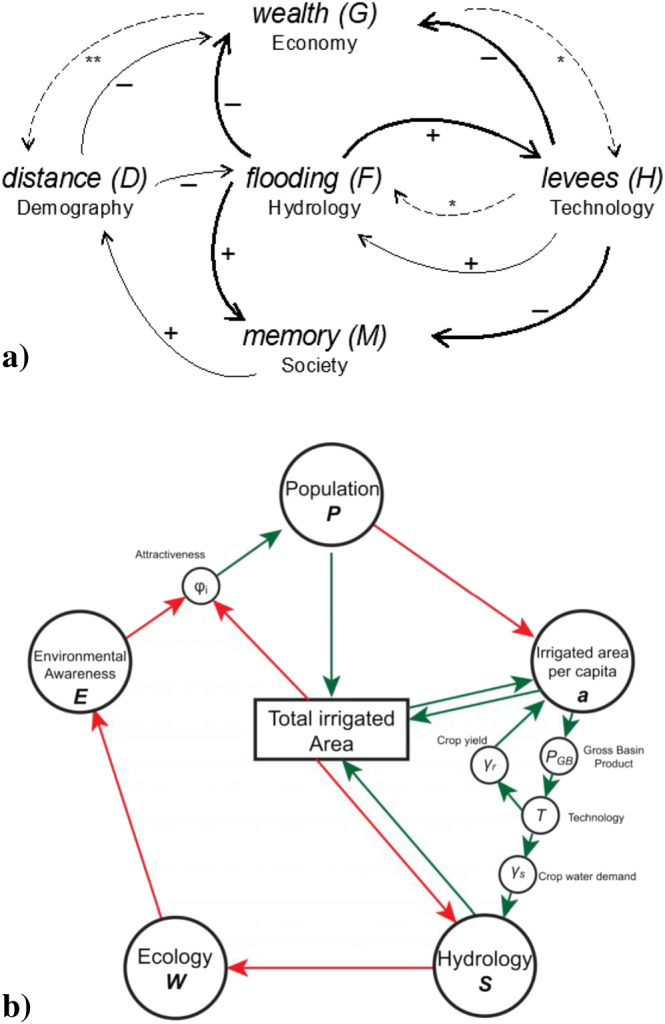
Examples of socio‐hydrological models as hypotheses about the feedback mechanisms generating one or more phenomena: (a) generic conceptualization of human‐flood interactions (Di Baldassarre, Viglione, et al., 2013) and (b) coupled human‐water dynamics in Murrumbidgee River basin (Van Emmerik et al., 2014).

Most early models of sociohydrology have been based on the system dynamics approach; see Blair and Buytaert (2016) and Troy, Pavao‐Zuckerman, & Evans, 2015, Troy, Konar, et al., 2015) for comprehensive reviews of recent socio‐hydrological models. These have been proposed as explanatory hypotheses about feedback mechanisms generating one or more observed classes of phenomena. The explanatory model depicted in Figure 3a, for example, is a (generic) system dynamics model, based on coupled differential equations (Di Baldassarre, Viglione, et al., 2013), that aims to explain, in a stylized manner, phenomena often observed in flood risk studies, that is, the aforementioned safe‐development paradox and adaptation effect. In the same way, Figure 3b depicts a (place‐based) conceptual model of the human‐water dynamics in the Murrumbidgee River basin in eastern Australia, including the competition between humans and the environment (Van Emmerik et al., 2014) that underlies the pendulum swing phenomenon. Similar place‐based models have been developed for the pendulum swing phenomena documented in Western Australia (Elshafei et al., 2014, 2016) and Tarim basin in western China (Liu et al., 2015).

The endogenization of human agency is the key to explain emergent phenomena (Pande & Sivapalan, 2017). To this end, different hypotheses have been proposed in sociohydrology. Di Baldassarre, Viglione, et al. (2013); Di Baldassarre et al. (2015) and Viglione et al. (2014), for example, built upon the concept of *social memory* (Folke et al., 2005) and explained the safe‐development paradox as resulting from a decay of flood memory during prolonged periods IN which flooding does not occur. In the same spirit, Van Emmerik et al. (2014), Liu et al. (2013), and Liu et al. (2015) explained the pendulum swing as the result of a competition between an economic productive force that favors human livelihoods and an environmental restorative force that favors the environment. As in Di Baldassarre, Viglione, et al. (2013), it was proposed that this competition was mediated by another social state variable, which Van Emmerik et al. (2014) termed an *environmental awareness*, and was later generalized by Elshafei et al. (2014) as *community sensitivity*. These social variables (e.g., social memory, community sensitivity) therefore played a central role in the development of associated coupled socio‐hydrological models. Elshafei et al. (2014), and subsequently Elshafei et al. (2016), provided an avenue for generalization of community sensitivity by connecting it to broad socio‐economic and socio‐cultural factors (e.g., human development index and corruption perception index). Roobavannan et al. (2017) further enhanced the power of community sensitivity by making it a function of the structure of the regional economy. The resulting suite of system dynamics models using either social memory or community sensitivity as a key state variable to explain the emergence of socio‐hydrological phenomena in effect have helped to endogenize human behavior and the feedbacks with the water system through deterministic human response relationships. For example, the migration of people out of, or toward, the Murrumbidgee River basin to or from other parts of eastern Australia was inspired by a law similar to Fick's law of dispersal (i.e., migration flux is proportional to negative gradient of unemployment), while community sensitivity itself was defined as a trade‐off between environmental health and economic well‐being. Migrants are often driven by their expectation of improved employment or earnings (Mabogunje, 1970; Todaro, 1969). Aspirations of better lives are based on household level decisions to either maximize expected income or minimize risk that the household is exposed to by diversifying the portfolio of income generating activities (Massey et al., 1993; Akay et al., 2012). Yet the decisions to migrate are often limited by substantial social and economic barriers (Bryan et al., 2014). As a result, it is often the individuals whose income is above average that migrate (Knight & Gunatilaka, 2010). The effect of natural hazards such as droughts and flooding can therefore be ambiguous (Chen et al., 2017; Gray & Mueller, 2012). On the one hand, it can reduce migration by removing resources necessary for migration to overcome set up costs or increasing labor demand in originating areas, while in some other cases it may reduce all income generating possibilities, pushing migrants *en masse* out of affected areas (Chen et al., 2017). Therefore, while migration may appear to respond to unemployment gradient (Roobavannan et al., 2017), it is much more complex phenomenon that deserves closer scrutiny.

There have also been early efforts to generalize from conclusions based on place‐based studies. One of the ways to achieve this is to invoke (either explicitly or implicitly) existing economic or sociological theories to propose alternative hypotheses about the feedback mechanisms that contribute to the emergence of socio‐hydrological phenomena. For example, Roobavannan et al. (2018) compared the outcomes of a socio‐hydrologic model of community sensitivity (Elshafei et al., 2014, 2016) with an independent analysis of proxy data (i.e., references to concerns about the environment appearing in Australian newspapers over a 100‐year period) carried out by Wei et al. (2017). Based on this analysis they argued that the concept of community sensitivity is consistent with the *values beliefs norms* theory widely adopted in sociology. Other socio‐hydrological modeling studies have postulated human behavior as one that maximizes a livelihood objective. Pande et al. (2011), for example, modeled basin‐scale water allocation based on *profit maximization* in agricultural production. Pande and Ertsen (2014) and Pande et al. (2014) provided an interpretation of the rise and dispersal of societies using *endogenous growth theory*, wherein actions of humans in maximizing their well‐being result in the formation of grander coalitions (i.e., rise of civilizations) and technological progress that accelerates both growth and environmental degradation. Grames et al. (2016) provided an economic, albeit profit maximizing interpretation of the safe‐development paradox.

While these models assumed that humans are consistently able to compare and contrast alternative bundles of goods and services and maximize their well‐being based on it (i.e., that they are rational), other approaches have been recently used to model *apparently* irrational behavior at individual and collective levels. Di Baldassarre et al. (2017), for instance, developed a system dynamics model by capturing *cognitive biases* at individual level in the management of droughts and floods, inspired by the idea of the availability heuristic in behavioral economics (Gal, 2018; Kahneman & Tversky, 1979). Yu et al. (2017) used *evolutionary game theory* to model the evolution of informal rules or norms of a community and associated collective action dynamics related to levee maintenance. Their model captures the social dilemma of how individually rational behavior can lead to collectively irrational outcome of poor levee maintenance as well as how the removal of short‐term flooding can lead to erosion of people's compliance to informal rules that regulate the social dilemma and, ultimately, erosion of community resilience to floods. Finally, Gunda et al. (2018) investigated the water stress response of the *Valdez acequia* in New Mexico (a community‐managed irrigation system) by linking a hydrological model to the system dynamics model of an *acequia* developed by Turner et al. (2016). They focused on the role that community social structure, in particular *mutualism*, plays in the ability of the *acequia* to maintain its functionality. They found that, while agricultural productivity declined, the community was able to maintain its functionality under streamflow declines due to adaptations like shifting crop selection.

The engagement with existing sociological and economic theories, and the development of new ones specific to sociohydrology, to provide explanations of observed socio‐hydrologic phenomena is important to ascertain whether these are exceptional dynamics occurring in particular places or are generic ones that can be extrapolated to other places or circumstances. However, they take on added significance in the context of the SDGs since, in the absence of previous history, we will be expected to drive policy choices within which water resource development can be kept in the *safe operating space for humanity* (Rockström et al., 2009).

Since coupled human‐water systems are complex systems that involve dynamics at multiple levels of human organization (Sivapalan & Blöschl, 2015), multiple levels of theories are likely to be needed to more fully understand socio‐hydrological phenomena. At individual level, promising theories of human behavior that remain insufficiently exploited in sociohydrology are the expected utility theory (Neumann & Morgenstern, 1944), protection motivation theory (Rogers, 1975), prospect theory (Kahneman & Tversky, 1979), game theory (Morrow, 1994), path dependency (Mahoney, 2000), and rebound effect or Jevon's paradox (Alcott, 2005). At collective level, applicable theories for explaining social change include collective action theory (Olson, 1965), institutionalist thinking (Cleaver, 2017; Ostrom, 1990; Van Laerhoven & Ostrom, 2007), and cultural evolution (Boyd & Richerson, 1985). Finally, at systems level, relevant theories could include resilience thinking (Walker et al., 2004), complex adaptive systems (Mitchell, 2009), metabolism theory (Banavar et al., 2002), and feedback control system theory (Doyle et al., 1990).

### 2.3 Reconceptualizing Water and Society Relations in the Context of SDGs

According to several scholars working across disciplines, the United Nations SDGs mark a paradigm shift in the way nature and society relations are understood and addressed in human development. First, the SDGs promote the notion of coupled human‐nature systems in which poverty reduction and planetary health are seen as intrinsically intertwined (Bello, 2013; Death & Gabay, 2015; Griggs et al., 2013; Langford, 2016; McMichael, 2017; Schleicher et al., 2018). It is argued that their transformative potential can only materialize if trade‐offs and the relation between poverty and environmental degradation are made explicit (Schleicher et al., 2018). Second, the three dimensions of sustainability have been presented as an *indivisible whole* and scholars warn that a fragmented implementation would generate perverse outcomes (Morton et al., 2017; Nilsson et al., 2016).

The nexus and trade‐offs within the SDGs, however, have only been superficially explored in the past. If on the one hand enhancing policy coherence for sustainable development is a key concern in the SDGs project (Target 17.14.1), on the other hand most complex and politically contentious trade‐offs have been glossed over in the international negotiations (Nilsson et al., 2016). Consequently, the multiple ways in which goals and dimensions of sustainability are interdependent are not explicitly discussed (Karlsson‐Vinkhuyzen et al., 2018). As aptly illustrated by Alcamo (2019: 126), *acting on synergies and trade‐offs requires not only political will but also knowledge about their origin and characteristics*. The risk, therefore, is that policy makers and bureaucrats end up working in vertical silos rather than through horizontal integrative approaches (Nilsson et al., 2016; Vandemoortele, 2011). Increasingly, scholars call for enhancing empirical knowledge on trade‐offs and synergies that can inform SDGs implementation processes. The UN's report *Mainstreaming of the three dimensions of sustainable development throughout the United Nations system* (UN Economic & Social Council, 2016) places water as a key integration force and highlights the relationship between multiple goals.

Sociohydrology can play an important role in conceptualizing SDG trade‐offs and feedback loops in the context of water‐society relations at different temporal and spatial scales. In section 2.2 we have shown how the body of literature that relates to sociohydrology has already theorized several phenomena that are relevant to the implementation of the SDGs. In the section that follows, we discuss opportunities to enrich sociohydrology by broadening its scope to address other water management dimensions relevant to the SDGs.

### 2.4 Expansion of Socio‐Hydrologic Phenomena: Broadening the Scope of SocioHydrology

Socio‐hydrologic phenomena taken up for study over the last 6 years have mostly examined human‐flood, human‐drought, and human‐environment interactions and feedbacks. Yet the role of water in SDGs extends well beyond these. For example, water resources are connected to food and energy production. Excessive exploitation of water to produce food and energy contributes to environmental degradation in some places. Hence, in addition to competition for water between humans and the environment, managing water in a broader context requires decisions about different human‐water uses (e.g., water vs food vs energy), or between different water hazards (e.g., floods vs droughts), both in time (e.g., short‐ vs long‐term considerations) and in space (e.g., upstream vs downstream and urban vs rural). Below, we focus on three examples: water pollution and human health, water‐energy‐food nexus, and transboundary water management.

#### 2.4.1 Water Pollution and Human Health

Globally, population growth and economic development have contributed to increased contamination (e.g., heavy metals, pharmaceuticals, pesticides, and fecal matter) of water supplies and of health risks related to waterborne diseases (Bain et al., 2014; Liu, Zhang, et al., 2017; Ternes et al., 2015). Although research on drinking water has overwhelmingly focused on use and access, a number of studies have examined contamination and its societal consequences and feedbacks at different scales. Work on the effects of coupled human‐water systems on drinking water quality includes research on the arsenic crisis in South East Asia and Bangladesh, respectively, analyzing hydrology of geogenic arsenic groundwater and health implications (Winkel et al., 2008; Michael & Voss, 2008; Sultana, 2011), and the role of power and gendered relations in shaping access to contaminated water (Sultana, 2006). Another example is recent research on intermittent water supply, which has demonstrated how *Escherichia coli* contamination is more likely to occur in areas where supply is not continuous (Agathokleous & Christodoulou, 2016; Kumpel & Nelson, 2013). Over 300 million people globally are served by intermittent water supply, and those who live in areas with inadequate sanitation are at higher risk of drinking contaminated water (Kumpel & Nelson, 2016; Sarpong Boakye‐Ansah et al., 2016). Risks of contamination are further exacerbated by storage practices adopted by residents to cope with discontinuity (Burt & Ray, 2014; Rusca et al., 2017). Improved sources are, thus, not necessarily free of pathogens and parasites and can cause waterborne diseases (Bain et al., 2014; Ercumen et al., 2015; Shaheed et al., 2014; Tosi Robinson et al., 2018).

Although these studies have identified technical challenges and household coping strategies that might lead to contamination and the health implications thereof, in the context of the SDGs more work can be done to unravel the interplay between the political economy of water and sanitation services, water contamination at different scales, and distribution of its risks. A growing body of literature proposes that safe water has become a commodity and calls for examining the relation between deterioration of water quality, class, gender, race, and indigenous rights to water (Rusca, Alda‐Vidal, et al., 2017,Rusca, Boakye‐Ansah, et al., 2017; Dodman et al., 2017; Vandewalle & Jepson, 2015). These studies suggest that there is a trend of vulnerable and marginalized communities suffering the most from exposure to unsafe drinking water. Sociohydrology can contribute by capturing more explicitly the dynamics generated by the interweaving of human and water‐quality transformations and the uneven distribution of risk through research on feedback loops between wastewater/sludge flows and society, including the economic, cultural, engineering, and human behaviors surrounding its mitigation and production at different scales.

The recent water crisis in Flint (Michigan, USA), for instance, mostly affected urban dwellers of more economically depressed background. The study by Butler et al. (2016) importantly shows the relationship among urbanization, economic development, inequalities, politics of water management, and water contamination. Flint developed mostly because of a *thriving* motor industry, while toxic industrial effluents contaminated the Detroit River. The ending of industrial activities left the city impoverished and, more recently, under emergency management to deal with the economic hardship. When, in order to reduce costs, the water supply was temporarily switched from Lake Huron to the Detroit River, residents were exposed for over 18 months to lead contaminated water, which caused elevated blood lead levels in residents, with irreversible impacts on children (Hanna‐Attisha et al., 2016; Liu, Zhang, et al., 2017). As for the pendulum swing phenomenon discussed above, economic and industrial development in the short term caused surface water contamination and environmental degradation in the long term. Ultimately, human actions led to contamination of drinking water, which compromised the health of residents.

One promising approach to capture sociohydrology of unsafe drinking water and its uneven distribution is developing interdisciplinary case studies that account for both the hydrological and social dimensions of water‐quality transformations. The aforementioned study by Rusca, Boakye‐Ansah, et al. (2017), for instance, drew attention to the interdependencies between socio‐political processes and microbiological and physiochemical contamination of drinking water and proposes a methodological approach to capture these (see also Boakye‐Ansah et al., 2016). Concurrently, sociohydrology may further understandings of risk perception in relation with water contamination and how extreme events may influence it. For instance, polls following the Flint water crisis show approximately half of the American population distrusts their tap water (Baum et al., 2016). These perceptions may, in turn, affect the way in which people access and consume water. All together these lines of inquiry have the potential to contribute to advancing SDG 6, as well as SDG 3 on good health and well‐being, especially ending waterborne diseases by 2030 (Target 3.3).

Last, synergies and trade‐offs involving water quality extend well beyond Goal 6. Alcamo (2019) points to some important and less explored trade‐offs: the expansion of conventional agriculture may contribute to Goal 2 (Zero hunger) but is likely to increase water pollution downstream; on the other end, high‐saline‐polluted water is inadequate for irrigation. Wastewater treatment is an energy intensive activity and will negatively impact Goal 7 (affordable and clean energy, see also Yillia, 2016). Today, at least one third of the rivers in Africa, Latin America, and Asia is polluted by untreated wastewater (United Nations Environment Program, UNEP, 2016). Wastewater treatment is, therefore, likely to have a major impact on the conservation, restoration, and sustainable use of terrestrial and inland freshwater ecosystems (Target 15.1). Last, as good water quality is a prerequisite of subsistence, it plays an important role in targets such as slum upgrading (Target 11.1) and reduction of inequalities (SDG 10) (Alcamo, 2019).

#### 2.4.2 Water‐Energy‐Food Nexus

Water plays a key role in several SDGs (as seen in Figure 2), and therefore, water management must account for these multiple interacting objectives, not just focusing on clean water and sanitation (UN Water, 2018). For example, water is at the heart of current research in the water‐energy‐food nexus across all spatial scales of analysis (Cudennec et al., 2018; D'Odorico et al., 2018; Lant et al., 2018; Liu, Yang, et al., 2017). A study by Yillia (2016) reveals the multiple trade‐offs and interconnections within this nexus. He suggests that interdependencies between water and energy have mostly focused on how few elements interact, rather than taking a more holistic and comprehensive approach. The interactions, she argues, are multiple and take place at different scales. These range from the use of water (SDG 6) to extract and transport energy sources, produce and convert energy, irrigate crops for biofuel production, and water intensive renewable energy (SDG 8). Energy is needed for treatment, distribution, and transport of drinking water and desalination (SDG6), as well as for irrigation (SD2). Last, as mentioned above, wastewater treatment is an energy intensive activity that plays a key role in ensuring water quality (SDG 6) and ecosystem protection (SDG 15).

Much research has been devoted to evaluating the vast water resources virtually embedded in internationally and regionally traded commodities, including food and energy (Allan, 2011; Hoekstra & Mekonnen, 2012; Konar et al., 2011; Marston et al., 2018; Wang et al., 2017). Future sociohydrology research may strive to represent these virtual flows of water in order to understand the full producer‐consumer chain of economic goods that require water (Konar et al., 2016). This will enable consumers to identify the water resources infrastructure that is supporting their food and energy receipts, enabling better accounting across SDGs. Critically, resolving these flows will enable consumers to better assess the exposure of their supply chains to water stress and shocks.

During the 2008 annual meeting of the World Economic Forum, participants called for a better understanding of the relationship between economic growth and (virtual) water flows (WEF Water Initiative, 2011). Causal inference research in economics has shown that international trade leads to increased economic growth (Frankel & Romer, 1999). Virtual water flows are essentially a rescaled metric of international commodity trade (typically provided in mass [tons] or value [$] units). It is therefore likely that virtual water trade also drives economic growth, though future research is to confirm this. Relatedly, recent causal inference work by Dang and Konar (2018) has shown that trade openness reduces the amount of water that the countries use in agriculture, with no impact to the industrial or total water use of nations. This research shows the average impact of trade on water use. Future research may explore the implications of trade for water use in specific settings and time domains.

This work by Dang and Konar (2018) highlights the interconnected nature of food‐energy‐water systems and illustrates that policies in one system may have spillover effects to another (e.g., economic trade policy has unintended consequences for water use in agriculture). Similarly, recent research by Deryugina and Konar (2017) employed causal inference methods to understand the impact of crop insurance on water use. They find that crop insurance leads to more irrigation withdrawals in agriculture, mainly due to farmers deciding to grow more water‐intensive crops. Thus, these studies highlight that policies that are supposedly unrelated to water (e.g., trade and crop insurance) can have unexpected consequences in terms of water consumptions. Similarly, the International Water Management Institute highlights how working to achieve the food SDG can have spillover effects for water (International Water Management Institute, 2018). This indicates that the SDGs are interrelated, and efforts to work toward the goal of one SDG may have unintended consequences for other SDGs. Future sociohydrology research should aim to better understand the complex interactions that exist in the food‐energy‐water nexus to enhance our ability to address all SDGs.

#### 2.4.3 Transboundary Water Management

Different SDGs give rise to different fundamental questions. Underlying questions concerning international basins is, as illustrated by Petersen‐Perlman et al. (2017, p. 13), the idea that *being part of a transboundary basin makes a user interconnected with the rest of its users*. For example, specific measures to promote economic growth (SDG 8) in a given place, for example, building large water infrastructure for food or energy production, can jeopardize other objectives in other places, such as reducing inequalities (SDG 10) or protecting and restoring water‐related ecosystems (SDG 15). In this perspective, cooperation in transboundary rivers (SDG6) can be also seen as one of the major environmental challenges of the century (Song & Whittington, 2004). This is particularly true in transboundary rivers that flow across political boundaries (De Stefano et al., 2017; Grey & Sadoff, 2003). The riparian states usually value water differently, leading to differences in water management regime, priorities, and culture. Consequently, transboundary river systems are characterized by both conflict and cooperation (Petersen‐Perlman & Wolf, 2015; Yoffe et al., 2003; Zeitoun & Mirumachi, 2008).

Conflict and cooperation in transboundary rivers can be seen as socio‐hydrological phenomena driven by the interplay of hydrological, technical, and social processes. They remain widely underresearched and not well understood. For example, it was found that no single indicator, be it climate, water scarcity, government type, or water demand, could explain the emergence of either conflict or cooperation (Yoffe et al., 2003). Hence, sociohydrology can contribute to a better understanding of cooperative and noncooperative responses by interpreting them as outcomes of long‐term coevolution (Pande & Ertsen, 2014), or as the effect of single events, such as the construction of the large dam or the occurrence of a climatic shock (Gleick, 2014). Therefore, taking a socio‐hydrological approach to transboundary water management can help to unravel the feedback mechanisms that explain these phenomena (Mianabadi et al., 2015) and, in turn, to meet water and environment‐related SDGs.

## 3 Scientific Challenges in Integrating the Social and Hydrological Dimensions

As sociohydrology strives to make headway toward improved understanding of the phenomena reviewed in sections 2.1 and 2.2 and broadens in scope to cope with a wider range of phenomena, including new phenomena that are likely to arise in the process of aiming to meet the SDGs, new methodological challenges arise. This section presents these challenges and outlines possible ways forward toward confronting these challenges.

### 3.1 Mechanisms and Phenomena Across Temporal and Spatial Scales

**Figure 4:**
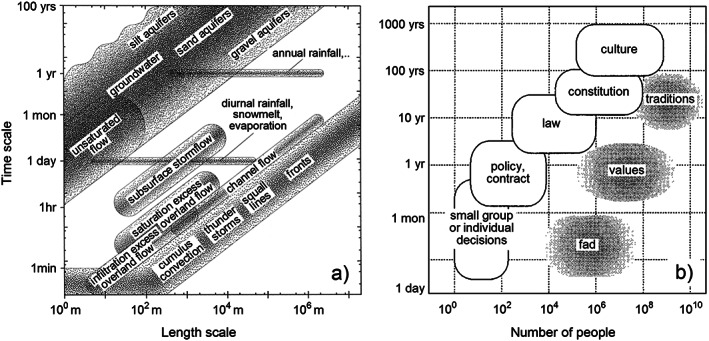
Temporal and spatial scales in (a) hydrological processes (Blöschl & Sivapalan, 1995) and (b) institutions (Gunderson & Holling, 2001).

One of the major challenges for sociohydrology is that the interplay of human and water systems involves both hydrological processes and social processes (and institutions) operating at multiple spatial and time scales (Figure 4). Capturing how these processes interact across time and space scales, for example, how short‐term processes affect longer term processes, and vice versa can provide important insights on the possible short‐ and long‐term impacts of implementing the SDGs and on spatial distribution of these impacts.

#### 3.1.1 Temporal Dynamics and Time Scale Interactions

Many of the phenomena discussed in section 2, such as the safe development paradox and the pendulum swing, tend to operate over long (decadal or centennial) time scales, but they often involve short‐term processes, such as the seasonal cycle of irrigation, or flood inundation and defense activities that happen within hours. Flood formation is coupled with event scale precipitation and seasonal soil moisture as well as with soil, vegetation, landscape, and climate evolution occurring over centuries to millennia (Gaál et al., 2012). Similarly, in human‐water systems, natural resources will slowly degrade if overused by human activities, such as water extraction or land use changes that do not allow for recovery (Ward et al., 2018). For example, large‐scale deforestation in Western Australia for agriculture caused shifts to the water balance, leading to slowly rising water tables and extensive land and stream salinization, which contributed to a decline in agriculture and reduction of human population (Elshafei et al., 2014, 2016).

The infrastructure system, typically, follows a slow evolutionary path linked to innovation through the interplay between technology and society (Geels, 2005; Pande et al., 2014). Technology includes infrastructure development to exploit water resources (e.g., irrigation) or improved water use efficiency in agriculture (Kandasamy et al., 2014), or river training and construction of levees to protect cities from flooding (Di Baldassarre, Kooy, et al., 2013). However, infrastructure develops in response to accumulated effects of human‐water interactions that occur at short time scales, for example, frequent flooding in urban communities can lead to raising or strengthening levees, likewise frequent water shortages in agricultural communities can lead to expanding water storage via reservoirs.

Depending on what time scales are considered, different feedbacks may become relevant (Blöschl & Sivapalan, 1995). If short time scales are considered, slow processes will not change much and so can be assumed as fixed boundary conditions, while this is no longer the case over a longer time frame. This notion was recognized as early as 1890 by economist Marshall (1890). He considered a market period (hours) where goods produced for sale on the market, for example, in a fish market prices are taken as given, but quickly adjust to clear markets; over longer periods industrial capacity is taken as given but outputs, employment, and inputs of raw materials fluctuate; at even longer periods the stock of capital goods, such as factories and machines, will fluctuate; when looking at even longer time frames, technology, population trends, habits, and customs are all likely to change.

#### 3.1.2 Legacy Risks

For long‐term processes related to hydrological, technical, or social parts of the system, a long‐term perspective becomes extremely relevant, particularly if it is difficult (or expensive) to reverse any decisions that are made. Some of the phenomena (Table 1) make it difficult for actors to reverse decisions when lock‐in situations occur. Such lock‐in situations may be economically, politically, or culturally driven. For example, due to the safe‐development paradox, or levee effect, once people have moved into floodplains it may be politically and economically difficult to relocate them to safer ground. Due to the irrigation efficiency paradox or economic rebound effect, a lock‐in situation occurred in India, where smallholders pumped groundwater at no cost as a result of subsidized energy. This measure is difficult to reverse as it enhances the stability of the elected provincial government (Shah et al., 2012). The supply‐demand cycle may result in lock‐ins due to high investment costs and may make societies dependent on expensive infrastructure, such as reservoirs for drought management (Di Baldassarre, Wanders, et al., 2018; Kuil et al., 2018, 2019), and eventually may result into a negative spiral toward peak water limits (Gleick & Palaniappan, 2010).

The importance of decisions over long time frames for these types of processes is reflected in the concept of *legacy risk*, which is the expected cost of decisions made today carried over a very long time frame. Traditionally, the concept of legacy risk originated in the domains of nuclear waste and mining as these are processes that may linger over very long time periods (Pepper et al., 2014; Russell, 2000). The concept, however, applies to a range of processes, including environmental management (Winiwarter et al., 2016). The idea is to put particular emphasis in today's planning on those decisions that may have enormous adverse effects for generations to come and will be difficult to reverse. The choice is a difficult one as *legacy risks the nation chooses not to bear cannot be separated from the opportunity costs (benefits given up elsewhere) it willingly incurs in reducing them*. Both risks and costs are *defined by inhabitants' values and preferences (and thus culture) and are not restricted to narrow, objective measures* (Russell, 2000, p. 4). Over and above this, there is also the issue of equity in relation to intergenerational and intragenerational distribution of risks and costs of these developments, as well as equity between different sectors of society (Zwarteveen et al., 2017; Zwarteveen & Boelens, 2014).

#### 3.1.3 Spatial Processes and Space‐Scale Interactions

Water‐related issues have a spatial component, which shapes trade‐offs, compromises, and negotiations between conflicting interests in different parts of the river basin (Loucks et al., 2005). In the water system, the spatial component arises because of spatial linkages of the water cycle. These linkages occur at all spatial scales, from the hillslope (through later surface and subsurface flow) right to the continental scale (through routing of water in river systems), and even globally (through atmospheric water transport) (Pringle, 2003; Savio et al., 2015; Van der Ent et al., 2010; Western et al., 1998). These spatial hydrological connectivities are relevant in terms of water quantity, such as upstream water uses that reduce downstream discharge; upstream flood retention that reduces downstream floods; water quality, that is, groundwater contamination affecting water quality further down the aquifer; and ecologically, such as in wetlands or at the river system scale.

These linkages also shape institutions, which interact at all spatial scales, from individuals to governments. Social stratification, power relationships, trust, cultural beliefs, and cognitive biases strongly influence the way in which different social groups can (or not) alter, perceive, and adapt to hydrological change. These heterogeneous elements also determine how water governance unfolds and, in turn, how water is managed at different scales (Pahl‐Wostl et al., 2013; Wei et al., 2017). As suggested by Zwarteveen et al. (2017), water governance is inherently political and concerns decisions on where and to whom water flows to and the institutions, norms, and beliefs underlying these decision‐making processes (see also Wei et al., 2017). Moreover, humans are *diverse, interpretive creatures who frequently disagree about values, means, and ends* (Castree et al., 2014: p.765; Massuel et al., 2018; Hulme, 2010).

Most phenomena discussed in section 2 can have a spatial component. For example, the pendulum swing manifested itself as a spatial process in the Kissimmee River basin in Florida (Chen et al., 2016). Severe flooding in the rural part of the catchment in the 1950s led to river training, which, however, degraded the local wetlands. The community in the urban part of the catchment valued the environment more than flood mitigation (as the latter did not concern them directly) resulting in river being remeandered and the wetlands being restored. The *safe‐development paradox*, or *levee effect*, very often has a spatial component when the construction of polders upstream of the floodplains under risk is considered. The irrigation efficiency paradox or rebound effect often has a spatial component in transboundary river management when water use increases due to regional water transfers (Dell'Angelo et al., 2018; Müller et al., 2016).

Socio‐hydrological entities are interconnected in today's highly connected world. The space‐time linkages are not only within each scale but also across scales and can lead to emergence of diverse phenomena such as large‐scale droughts. Trade networks spread knowledge and can bring in sudden changes in land use policies such as rapid deforestation in the Amazon or even changes in how resources are governed. The understanding of dynamic patterns of interconnectedness through trade and the global hydrological cycle is therefore critical to the assessment of long‐term water resource availability at global and local scales. This demands an extension of systems with endogenous human agency to space and to space‐time. A natural extension is therefore to endogenize the boundary conditions themselves, for example, of trade or rainfall, just as sociohydrology has endogenized scenarios in the time domain, for example, of population growth, through bidirectional feedbacks. This requires us to understand additional processes that connect socio‐hydrological entities in space, with the goal of explaining why aggregated, macroscopic behavior emerges from heterogeneous components.

### 3.2 Evaluating the Implications of the SDGs: Integrating Quantitative and Qualitative Data

One scientific challenge in sociohydrology is integrating qualitative reporting and case studies to provide in‐depth, context specific, critical descriptions of human‐water interactions at different scales (Massuel et al., 2018; Mostert, 2018; Sexsmith & McMichael, 2015; Weststrate et al., 2018). This approach can contribute to widen the parameters used to examine the effectiveness of SDGs and ensure aspects related to human psychological, physical, and emotional well‐being are taken into consideration.

A second reason for integrating quantitative and qualitative data is to deepen the analysis of phenomena and processes that may enable or constrain the success of SDGs. This also entails critically examining the implementation of the water‐related SDGs by questioning the underlying political, ideological, and economic logics. Several authors have warned that no long‐term prosperity is possible without changes in the current economic system and that the new targets will not be effective if structural processes that perpetuate poverty are not addressed (Bello, 2013; Death & Gabay, 2015; Langford, 2016; Liverman, 2018). This interpretation shared by the UN report of the high‐level panel of eminent persons on the post‐2015 development, co‐chaired by Susilo Bambang Yudhoyono (at the time President of Indonesia), Ellen Johnson Sirleaf (at the time President of Liberia), and David Cameron (at the time Prime Minister of the United Kingdom), recognizes that it is *unrealistic to think we can help another one billion people to lift themselves out of poverty […] without making structural changes in the world economy* and calls for new models (United Nations, UN, 2013: p.5).

Vandemoortele (2011, p. 13), one of the architects of the Millennium Development Goals, claimed that they *have been misappropriated to reaffirm the conventional view of development*. SDGs need to propose a new vision and a new understanding of sustainability, economic growth, and the environment, recognizing that *the global economy services society, which lies within Earth's life‐support system* (Griggs et al., 2013: 305; see also Bello, 2013). In other words, this requires a transformative change in the ways that economic development is conceived (sustainability) and financed (accountability and priority setting) and the benefits thereof are distributed (equity, Mawdsley, 2018; Mawdsley, 2017; Sexsmith & McMichael, 2015). A productive engagement between sociohydrology and critical resource geography may produce relevant insights on the relation between financial flows, power, and hydrological flows in the implementation of the water‐related SDGs at different temporal and spatial scales, as well as on how the benefits and costs of these developments are distributed between societal groups and across spaces. In doing so, sociohydrology has the potential to contribute more effectively also to goals focusing on poverty (SDG 1) and equity (SDG 10).

These examinations, we argue, require both integrating qualitative approaches *alongside* quantitative analyses and *within* modeling exercises to better understand the current socio‐political, economic, and cultural contexts in different locations and how these contribute to co‐shape SDG outcomes. Given its theoretical paradigm, sociohydrology is well positioned to play a catalytic role in integrating qualitative and quantitative data, yet this endeavor entails major scientific challenges.

A first challenge in doing this is offered by different epistemologies, research strategies, and axiologies of qualitative and quantitative approaches (Mostert, 2018; Romagny & Cudennec, 2006; Wesselink et al., 2016). To preserve the richness and epistemological perspective of qualitative approaches, Mostert (2018) suggests developing case studies on the multiple ways in which humans alter hydrology in specific contexts and carefully selecting cases for comparative analysis and, possibly, for generalization of some patterns (see, for instance, Robinson, 2016). In their study on drinking water quality in the city of Lilongwe, Rusca, Boakye‐Ansah, et al., 2017; Rusca, Alda‐Vidal, et al., 2017) propose a case study design that accounts for both the complexity of the human dimension(s) and material and quantitative variables of the hydrological system. This approach allows retaining central themes of scholars in critical water studies and political ecology such as the role of ideology, power, class, gender, culture, and everyday practices in controlling and directing water flows in rural, urban, and regional landscapes and the uneven distribution of costs and benefits thereof (Alda‐Vidal et al., 2018; Ekers & Loftus, 2008; Swyngedouw, 1997; Tiwale et al., 2018; Truelove, 2011; Truelove, 2016), and relating these to changes to the hydrological system.

A second specific challenge with integrating quantitative and qualitative data concerns how to capture aspects that are (i) heterogeneous (i.e., diverse for different social groups), (ii) typically described in a qualitative way, and (iii) rarely collected in a systematic fashion. For instance, by looking at the five variables of the loop diagram presented earlier in Figure 3a, one can see that while some of these variables can be easily quantified and are often available as systematic time series (e.g., flooding, expressed as high water levels above a datum; Di Baldassarre, Viglione, et al., 2013,Di Baldassarre, Kooy, et al., 2013; Di Baldassarre et al., 2015), others are more qualitative and typically not systematically available over time (e.g., flood memory, expressed as the level of risk awareness in a community; Di Baldassarre, Viglione, et al., 2013; Di Baldassarre, Kooy, et al., 2013; Di Baldassarre et al., 2015). Moreover, flood memory is heterogeneously accumulated in different individuals or social groups, and these various individuals or social groups have different capacities to influence the decision‐making process and to feed back on the hydrological system.

In socio‐hydrological modeling, the most common approach has been to turn qualitative information into quantitative using some proxies, such as flood insurance coverage as an analog of the level of flood risk awareness (Di Baldassarre et al., 2017), or using social media coverage, newspaper articles, or Google trends (Roby et al., 2018; Wei et al., 2017). Yet these short‐cut methods raise numerous concerns, such as limited representativeness and their unavoidable inability to describe the heterogeneity of human society. Thus, there remains a need to better integrate social and hydrological sciences as well as qualitative and quantitative information for the study of human‐water interactions.

### 3.3 Exploiting Socio‐Hydrologic Understanding to Enable Improved IWRM Practice

**Figure 5:**
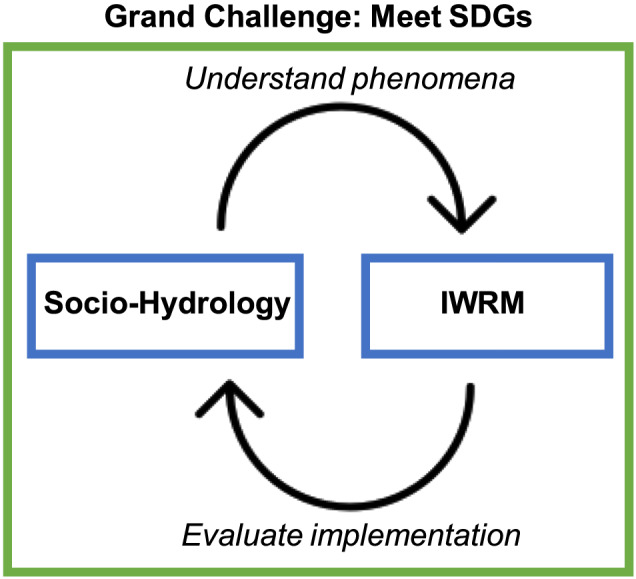
Conceptual framing of the relationships between sociohydrology, Integrated Water Resources Management (IWRM), and the Sustainable Development Goals (SDGs). Both sociohydrology and IWRM strive to achieve the SDGs. It is imperative that sociohydrology and IWRM communicate with each other and learn from one another to this end.

The advances expected to be made in socio‐hydrologic understanding raises the question as to how these can help advance water management practice, notably IWRM, in the context of the UN SDGs. Sociohydrology can play a role in working toward societal water goals in two major ways. First, sociohydrology aims to provide scientific insights that can be used to inform IWRM. This is the detection and understanding of phenomena that exist in water resources management (see above; sections 2.1 and 2.2). Both sociohydrology and IWRM aim to help achieve SDGs (Figure 5), particularly the water‐related targets highlighted in Figure 2. Second, sociohydrology is well‐suited to scientifically evaluate the consequences of IWRM implementation. For example, sociohydrology can integrate understanding from the policy evaluation community (e.g., causal inference) to assess whether or not water management or policy had the intended outcome. In this way, sociohydrology and IWRM should strive to inform one another and learn from each other in a *two way street* of information exchange (see Figure 5).

As discussed in section 2.1, several emergent phenomena have been established through sociohydrology research. These phenomena have clear implications for IWRM. In Table 1, we map how a better understanding of socio‐hydrological phenomena can provide useful insights to improve IWRM and help lead to desirable societal goals set forth by SDGs. For instance, through a better understanding of the rebound effect, we show that implementing drip irrigation alone will not lead to reduced water use, but, rather, may actually lead to increased water use. This means that IWRM should consider implementing counteracting measures, such as water basin use caps, along with irrigation efficiency technologies.

It is essential to evaluate whether an IWRM intervention actually leads to the desired outcome. For example, does installing improved sanitation in a local community actually lead to reductions in waterborne diseases and diarrheal outbreaks? Sociohydrology can lead the way in evaluating whether or not IWRM interventions have the desirable impacts. Sociohydrology can do this through the adoption of novel techniques from the policy evaluation and econometrics fields. In fact, this increased interaction between sociohydrology and economics was recently called for by Müller and Levy (2019). The tools of randomized controlled trials (RCTs) and causal inference using natural experiments in empirical information can be exploited to properly determine whether or not IWRM interventions are leading toward the desired societal goals or the SDGs.

When possible, RCTs should be used to determine the outcome of an intervention. For example, to address the question about improved sanitation implementation discussed above, it would be best to install improved sanitation facilities *randomly* in some locations and not install them in others and then assess the outcomes (Null et al., 2018). The key is that the treatment (e.g., improved sanitation) must be randomly assigned in order to determine its causal impact on the societal outcome of interest (e.g., diarrhea). RCTs are the gold standard for assessing the impact of a treatment. For this reason, whenever possible, randomization of IWRM management techniques or interventions would be the preferred course of action.

Unfortunately, it will often not be feasible to perform an RCT to evaluate IWRM action. This is because they are expensive, time consuming, and may sometimes be unethical. For this reason, exploiting naturally occurring data (i.e., nonexperimental) that has some attributes that can help us to distinguish that causal impact of the treatment on the outcome of interest is critical. This is the aim of causal inference in econometrics. Causal inference has recently been applied to understand how human interventions impact water resources, such as for crop insurance (Deryugina & Konar, 2017) and international trade (Dang & Konar, 2018). Going forward, it will be of continued importance for sociohydrology to employ causal inference tools to assess the performance of IWRM in helping us meet the SDGs.

### 3.4 Addressing a Societal Grand Challenge: Managing Trade‐Offs Between SDGs and Overcoming Legacy Risks

The water crises that humanity faces are increasingly multifaceted, complex, and intertwined. This is clearly reflected in the fact that many of the United Nations SDGs may affect or are affected by the quantity and quality of water resources (Figure 2). Linear and short‐term approaches to fix one problem in isolation often trigger new problems. Failure to anticipate and comprehensively address emergent water‐related risks of floods, droughts, and water‐quality degradation holistically and inclusively can cause economic decline and can lead some communities into a poverty trap, an emergent phenomenon highlighted by Borgeomo et al. (2018) based on work in Bangladesh. This shows how, at a broader level, water management is intimately tied to SDG 1, which aims at eradicating poverty.

Managing water‐related issues comprehensively and holistically thus requires nontrivial decisions about what to prioritize (e.g., drinking vs sanitation vs food), who pays to address these issues and how much, how the benefits and the costs of these measures are distributed (rich vs poor, upstream vs downstream, and urban vs rural), and how to balance benefits and costs (economic vs environmental vs social). It is essential to fully integrate water resources management across the entire socio‐hydrological cycle; different water users including engineering, economic, social, ecological, and legal aspects; and spatial scales, including, for example, upstream/downstream perspectives. While technical measures can work in the short term, the absent (or limited) involvement of society‐at‐large (i.e., the beneficiaries) often leads to adverse consequences such as resistance to (or nonacceptance of) introduced policies. Recognizing the nonlinear nature of human‐water systems and pursuing broader and long‐term perspectives are key steps to craft more robust decisions (Haasnoot et al., 2013) and tackle the current and future water challenges in a more sustainable way.

**Figure 6:**
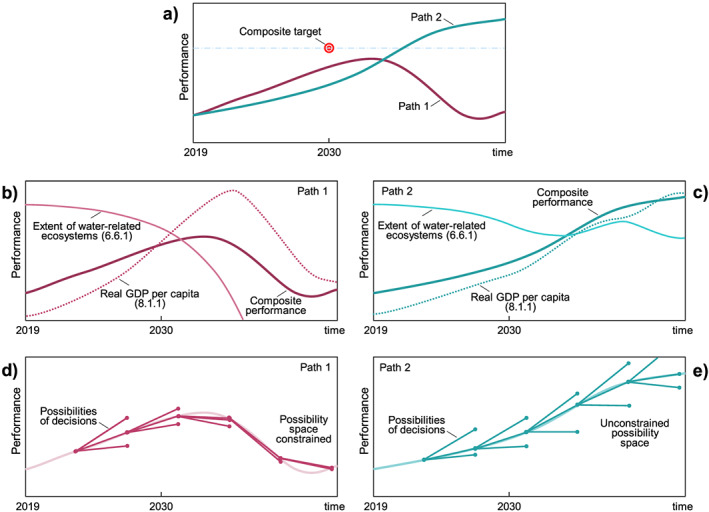
Addressing a societal grand challenge, for example, performance in achieving the SDGs. Conceptual drawings illustrating: (a) short‐term (Path 1) versus long‐term (Path 2) perspective. Path 1 gets closer than Path 2 to the composite target by 2030, which is the year in which SDGs are expected to be achieved, but Path 2 is more sustainable and more effective than Path 1 in the longer term. (b and c) Decompositions of Path 1 (short‐term) and Path 2 (long‐term) exemplifying how short‐term perspectives can become unsustainable in the long term. Path 1 relies on fast economic growth but with unsustainable environmental costs, that is, degradation of wetlands and aquifer depletion. (d and e) Possibility space and decision trees for Path 1 (constrained) Path 2 (unconstrained). These two panels show that water policies and management decisions are typically updated over time (dots in the diagrams). The possibility space of Path 1 becomes constrained (Figure 6d) once that water resources are depleted, as environmental degradation and economic decline are essentially irreversible. The possibility space of Path 2 remains unconstrained (Figure 6e), that is, policy and decision makers can keep influencing the trajectory over time and therefore adjust and adapt to, for example, climatic and socio‐economic changes.

Figure 6 illustrates, in a simple and schematized way, the variation over time of the performance in addressing societal challenges. It can be seen, for example, as a composite value of various indicators for the achievement of the SDGs. For the sake of simplicity, we consider only two of them (i) the real Gross Domestic Product (GDP) per capita (SDG8, Indicator for target 1.1); and the extent of wetlands over time (SDG 6, Indicator for target 6.6). In Figure 6a, we see that Path 1 gets closer than Path 2 to the composite target by 2030, which is the year in which SDGs are expected to be achieved. A short‐term approach would therefore favor policies associated with Path 1. However, Path 2 is more sustainable, and it is more effective than Path 1 in the longer term (Figure 6a). Figures 6b and 6c decompose these two paths and exemplify how short‐term perspectives can become unsustainable in the long term. Path 1 relies on fast economic growth but with unsustainable environmental costs, that is, degradation of wetlands and aquifer depletion. In the long term, not only does this narrow approach prevent further growth but it also leads to increasing poverty (Figure 6b). Path 2, instead, is promoting economic growth but without compromising the environment, depleting water resources, and compromising future generations. In the long term, this broader approach results more sustainable (Figure 6c).

Indeed, all of the socio‐hydrological phenomena discussed previously in section 2 can be seen as a *legacy*: the result of a coevolution between hydrological, technical, and social systems, which influence or constrain today's decision. As the past was a legacy to the present, the present is a legacy for the future. This implies that water policies and management decisions have a *legacy risk*. Short‐term fixes based on linear thinking can have negative repercussion in the long term, which might be difficult, if not impossible, to reverse, that is, a lock‐in condition. To cope with legacy risks, we need to look at future trajectories and exclude decisions that cannot be reversed, that is, not to constraining the future possibility space. Thus, understanding how the interactions and feedbacks between social, technical, and hydrological processes generate legacy in water management is crucial to reducing legacy risk. Figures 6d and 6e illustrate these concepts. Path 1 and Path 2 are represented using decision trees in the possibility space. This illustration reflects the fact that water policies and management decisions are typically updated over time. In the example of Figure 6d, the possibility space of Path 1 becomes constrained once that water resources are depleted, as environmental degradation and economic decline are essentially irreversible. Instead, the possibility space of Path 2 remains nonconstrained. Policy and decision makers can keep influencing the trajectory over time and therefore adjust and adapt to, for example, climatic and socio‐economic changes.

The concept of legacy risk ties in well with the precautionary principle. In its strongest formulation, the precautionary principle calls for absolute proof of safety before allowing new technologies to be adopted (Foster et al., 2000). It is an important component of much of the environmental legislation in the western world. The reasoning is similar to that of legacy risk, that is, to err on the side of least consequences. In fact, the concept of legacy risk can be interpreted as a dynamic version of the precautionary principle. When in doubt, the decisions we make must try to avoid those development paths that potentially have irreversible effects resulting from the types of phenomena discussed in this paper. The generalized understanding of phenomena that may lead to lock‐in situations, be they economically, politically, culturally, or environmentally driven, will provide guidance as to what extent the principle should be weighed against quantitative evidence, cost‐benefit analyses, and discretionary judgment. Work on time‐space scale interactions, the opportunities of using both qualitative and quantitative information, and setting up a more integrated interdisciplinary collaboration process will help in making progress in understanding generalizable phenomena. The levee effect, supply‐demand cycles, and the irrigation efficiency paradox are all reflections of coupled human‐water processes that may or may not constrain future decision making.

## 4 Conclusions

In this paper, we examined the role of sociohydrology in addressing the societal grand challenges posed by the SDGs. We argued that sociohydrology is an appropriate analytical framework to conceptualize and evaluate the water implications of the SDGs. First, sociohydrology can act as an interface between the SDGs and IWRM principles. In conceptualizing water challenges, sociohydrology considers socio‐cultural and socio‐political dimensions as well as short‐term and long‐term impacts of water governance processes. As such, it provides both actionable and generalizable insights on short‐ and long‐term impacts of the strategies adopted to meet the SDGs targets. Second, sociohydrology has focused on the explanation of a wide range of phenomena that may enable or constrain the success of water‐related SDGs. There is considerable scope for applying these insights to strategies developed by different countries to meet the SDGs. Last, in the coming years sociohydrology will critically examine the implementation of the water‐related SDGs to monitor their short‐ and long‐term sustainability. Concurrently, the paper identified a series of research directions to further sociohydrology and enhance its contribution to the SDGs. We propose a research agenda that widens the range of phenomena—including water pollution and health‐related challenges, the water‐food‐energy nexus, and transboundary water governance—and explores and tests innovative methodologies to trace water‐society interactions at different temporal and spatial scales.

To achieve the United Nations SDGs, there is a need to (i) capture the complex, multifaceted nature of human‐water systems and (ii) explore long‐term dynamics associated with alternative policies. These two aspects have been the very essence of socio‐hydrological research over the past 6 years. However, it is no longer adequate to investigate, *after the fact*, the causes of water crises in different contexts and frame them theoretically as the unintended consequences of water resources management or governance. Increasingly, sociohydrology must aspire to assist communities involved in IWRM to frame water‐related issues in broader terms and develop models capable of generating likely alternative futures under various policy options (Bai et al., 2016). This will empower communities, and in particular the IWRM community, to make more informed decisions for sustainable development and management of water resources. This requires a broadening of the theoretical foundations and methodological diversity of sociohydrology, going beyond individual phenomena, and toward mapping out the safe operating space for humanity to manage their water resources sustainably. By being open to face up to these scientific challenges, sociohydrology is well placed to help communities to meet the SDGs, the societal grand challenge of our time.

## References

[wrcr24091-bib-0001] Adger, W. N. (2006). Vulnerability. Global Environmental Change, 16(3), 268–281. 10.1016/j.gloenvcha.2006.02.006

[wrcr24091-bib-0002] Adger, W. N. , Quinn, T. , Lorenzoni, I. , Murphy, C. , & Sweeney, J. (2013). Changing social contracts in climate‐change adaptation. Nature Climate Change, 3(4), 330–333. 10.1038/nclimate1751

[wrcr24091-bib-0003] Agathokleous, A. , & Christodoulou, S. (2016). The impact of intermittent water supply policies on urban water distribution networks. Procedia engineering, 162, 204–211. 10.1016/j.proeng.2016.11.041

[wrcr24091-bib-0004] Akay, A. , Bargain, O. , & Zimmermann, K. F. (2012). Relative concerns of rural‐to‐urban migrants in China. Journal of Economic Behavior & Organization, 81(2), 421–441. 10.1016/j.jebo.2011.12.006

[wrcr24091-bib-0005] Alcamo, J. (2019). Water quality and its interlinkages with the Sustainable Development Goals. Current Opinions in Environmental Sustainability, 36, 126–140. 10.1016/j.cosust.2018.11.005

[wrcr24091-bib-0006] Alcott, B. (2005). Jevons' paradox. Ecological Economics, 54(1), 9–21. 10.1016/j.ecolecon.2005.03.020

[wrcr24091-bib-0007] Alda‐Vidal, C. , Kooy, M. , & Rusca, M. (2018). Mapping operation and maintenance: An everyday urbanism analysis of inequalities within piped water supply in Lilongwe, Malawi. Urban Geography, 39(1), 104–121. 10.1080/02723638.2017.1292664

[wrcr24091-bib-0008] Allan, T. (2011). Virtual water: Tackling the threat to our planet's most precious resource, (p. 384). London: I. B. Taurus.

[wrcr24091-bib-0009] Anderies, J. M. (2015). Managing variance: Key policy challenges for the Anthropocene. Proceedings of the National Academy of Sciences, 112(47), 14,402–14,403. 10.1073/pnas.1519071112 PMC4664313 26578808

[wrcr24091-bib-0010] Bai, X. , van der Leeuw, S. , O'Brien, K. , Berkhout, F. , Biermann, F. , Brondizio, E. S. , Cudennec, C. , Dearing, J. , Duraiappah, A. , Glaser, M. , Revkin, A. , Steffen, W. , & Syvitski, J. (2016). Plausible and desirable futures in the Anthropocene: A new research agenda. Global Environmental Change, 39, 351–362. 10.1016/j.gloenvcha.2015.09.017

[wrcr24091-bib-0011] Bain, R. E. , Cronk, R. , Wright, J. , Yang, H. , Slaymaker, T. , & Bartram, J. (2014). Fecal contamination of drinking‐water in low‐ and middle‐income countries: A systematic review and meta‐analysis. PLoS Medicine, 11(5), e1001644 10.1371/journal.pmed.1001644 24800926 PMC4011876

[wrcr24091-bib-0012] Banavar, J. R. , Damuth, J. , Maritan, A. , & Rinaldo, A. (2002). Supply–demand balance and metabolic scaling. Proceedings of the National Academy of Sciences of USA, 99(16), 10,506–10,509. 10.1073/pnas.162216899 PMC124956 12149461

[wrcr24091-bib-0013] Barendrecht, M. H. , Viglione, A. , Kreibich, H. , Merz, B. , Vorogushyn, S. , & Blöschl, G. (2019). The value of empirical data for estimating the parameters of a socio‐hydrological flood risk model. Water Resources Research, 55, 1312–1336. 10.1029/2018WR024128 31007299 PMC6472491

[wrcr24091-bib-0014] Baum, R. , Bartram, J. , & Hrudey, S. (2016). The Flint water crisis confirms that US drinking water needs improved risk management. Environmental Science and Technology, 50(11), 5436–5437. 10.1021/acs.est.6b02238 27187151

[wrcr24091-bib-0015] Bello, W. (2013). Post‐2015 development assessment: Proposed goals and indicators. Development, 56(1), 93–102. 10.1057/dev.2013.10

[wrcr24091-bib-0016] Blair, P. , & Buytaert, W. (2016). Socio‐hydrological modelling: A review asking "why, what and how?". Hydrology and Earth System Sciences, 20(1), 443–478. 10.5194/hess-20-443-2016

[wrcr24091-bib-0017] Blöschl, G. , Nester, T. , Komma, J. , Parajka, J. , & Perdigão, R. A. P. (2013). The June 2013 flood in the Upper Danube Basin, and comparisons with the 2002, 1954 and 1899 floods. Hydrology Earth System Sciences, 17(12), 5197–5212. 10.5194/hess-17-5197-2013

[wrcr24091-bib-0018] Blöschl, G. , & Sivapalan, M. (1995). Scale issues in hydrological modelling—A review. Hydrological Processes, 9(3‐4), 251–290. 10.1002/hyp.3360090305

[wrcr24091-bib-0019] Boakye‐Ansah, A. S. , Ferrero, G. , Rusca, M. , & van der Zaag, P. (2016). Inequalities in microbial contamination of drinking water supplies in urban areas: The case of Lilongwe, Malawi. Journal of Water and Health, 14(5), 851–863. 10.2166/wh.2016.258 27740550

[wrcr24091-bib-0020] Borgeomo, E. , Hall, J. W. , & Salehin, M. (2018). Avoiding the water‐poverty trap: Insights from a conceptual human‐water dynamical model for coastal Bangladesh. International Journal of Water Resources Development, 34(6), 900–922. 10.1080/07900627.2017.1331842

[wrcr24091-bib-0021] Boyd, R. , & Richerson, P. J. (1985). Culture and the evolutionary process. Journal of Nervous and Mental Disease, 175(2), 125–126.

[wrcr24091-bib-0022] Breyer, B. , Zipper, S. C. , & Qiu, J. (2018). Sociohydrological impacts of water conservation under anthropogenic drought in Austin, TX (USA). Water Resources Research, 54, 3062–3080. 10.1002/2017WR021155

[wrcr24091-bib-0023] Brown, C. (2007). Differential equations—A modelling approach, Series: Quantitative Applications in the Social Sciences (p. 120). Thousand Oaks, CA: SAGE Publications, Inc.

[wrcr24091-bib-0024] Brown, C. M. , Lund, J. R. , Cai, X. , Reed, P. M. , Zagona, E. A. , Ostfeld, A. , Hall, J. , Characklis, G. W. , Yu, W. , & Brekke, L. (2015). The future of water resources systems analysis: Toward a scientific framework for sustainable water management. Water Resources Research, 51, 6110–6124. 10.1002/2015WR017114

[wrcr24091-bib-0025] Bryan, G. , Chowdhury, S. , & Mobarak, A. M. (2014). Underinvestment in a profitable technology: The case of seasonal migration in Bangladesh. Econometrica, 82(5), 1671–1748.

[wrcr24091-bib-0026] Burt, Z. , & Ray, I. (2014). Storage and non‐payment: Persistent informalities within the formal water supply of Hubli‐Dharwad, India. Water Alternatives, 7(1), 106–120.

[wrcr24091-bib-0027] Burton, C. , & Cutter, S. L. (2008). Levee failures and social vulnerability in the Sacramento‐San Joaquin Delta area, California. Natural Hazards Review, 9(3), 136–149. 10.1061/(ASCE)1527-6988(2008)9:3(136)

[wrcr24091-bib-0028] Butler, L. J. , Scammell, M. K. , & Benson, E. B. (2016). The Flint, Michigan, water crisis: A case study in regulatory failure and environmental injustice. Environmental Justice, 9(4), 93–97. 10.1089/env.2016.0014

[wrcr24091-bib-0029] Cash, D. W. , Adger, W. N. , Berkes, F. , Garden, P. , Lebel, L. , Olsson, P. , Pritchard, L. , & Young, O. (2006). Scale and cross‐scale dynamics: Governance and information in a multilevel world. Ecology and Society, 11(2), 8.

[wrcr24091-bib-0030] Castree, N. , Adams, W. M. , Barry, J. , Brockington, D. , Büscher, B. , Corbera, E. , Demeritt, D. , Duffy, R. , Felt, U. , Neves, K. , Newell, P. , Pellizzoni, L. , Rigby, K. , Robbins, P. , Robin, L. , Rose, D. B. , Ross, A. , Schlosberg, D. , Sorlin, S. , West, P. , Whitehead, M. , & Wynne, B. (2014). Changing the intellectual climate. Nature Climate Change, 4(9), 763–768. 10.1038/nclimate2339

[wrcr24091-bib-0031] Castro, J. E. (2007). Water governance in the twentieth‐first century. Ambiente & Sociedade, 10(2), 97–118. 10.1590/S1414-753X2007000200007

[wrcr24091-bib-0032] Chang, H. , Thiers, P. R. , Netusil, N. R. , Yeakley, J. A. , Rollwagen‐Bollens, G. , Bollens, S. , & Singh, S. (2014). Relationships between environmental governance and water quality in a growing metropolitan area of the Pacific Northwest, USA. Hydrology and Earth System Sciences, 18, 1383–1395. 10.5194/hess-18-1383-2014

[wrcr24091-bib-0033] Chen, J. J. , Mueller, V. , Jia, Y. , & Tseng, S. K. H. (2017). Validating migration responses to flooding using satellite and vital registration data. American Economic Review, 107(5), 441–445. 10.1257/aer.p20171052

[wrcr24091-bib-0034] Chen, X. , Wang, D. , Tian, F. , & Sivapalan, M. (2016). From channelization to restoration: Socio‐hydrologic modeling with changing community preferences in the Kissimmee River Basin. Water Resources Research, 52, 1227–1244. 10.1002/2015WR018194

[wrcr24091-bib-0035] Ciullo, A. , Viglione, A. , Castellarin, A. , Crisci, M. , & Di Baldassarre, G. (2017). Socio‐hydrological modelling of flood‐risk dynamics: Comparing the resilience of green and technological systems. Hydrological Sciences Journal, 62(6), 880–891. 10.1080/02626667.2016.1273527

[wrcr24091-bib-0036] Cleaver, F. (2017). Development through bricolage: Rethinking institutions for natural resource management, (p. 224). London: Routledge.

[wrcr24091-bib-0037] Cleaver, F. , & De Koning, J. (2015). Furthering critical institutionalism. International Journal of the Commons, 9(1), 1–18. 10.18352/ijc.605

[wrcr24091-bib-0038] Cosens, B. , Gunderson, L. , & Chaffin, B. (2018). Introduction to the special feature practicing panarchy: Assessing legal flexibility, ecological resilience, and adaptive governance in regional water systems experiencing rapid environmental change. Ecology and Society, 23(1), 4 10.5751/ES-09524-230104

[wrcr24091-bib-0039] Csete, M. E. , & Doyle, J. C. (2002). Reverse engineering of biological complexity. Science, 295(5560), 1664–1669. 10.1126/science.1069981 11872830

[wrcr24091-bib-0040] Cudennec, C. , Liu, J. , Qi, J. , Yang, H. , Zheng, C. , Gain, A. K. , Lawford, R. , de Strasser, L. , & Yillia, P. T. (2018). Epistemological dimensions of the water‐energy‐food nexus approach. Hydrological Sciences Journal, 63(12), 1868–1871. 10.1080/02626667.2018.1545097

[wrcr24091-bib-0041] Dang, Q. , & Konar, M. (2018). Trade openness and domestic water use. Water Resources Research, 54, 4–18. 10.1002/2017WR021102

[wrcr24091-bib-0042] Daniel, D. , Marks, S. J. , Pande, S. , & Rietveld, L. (2018). Socio‐environmental drivers of sustainable adoption of household water treatment in developing countries. npj Clean Water, 1(1). 10.1038/s41545-018-0012-z

[wrcr24091-bib-0043] De Stefano, L. , Petersen‐Perlman, J. D. , Sproles, E. A. , Eynard, J. , & Wolf, A. T. (2017). Assessment of transboundary river basins for potential hydro‐political tensions. Global Environmental Change, 45, 35–46. 10.1016/j.gloenvcha.2017.04.008

[wrcr24091-bib-0044] Death, C. , & Gabay, C. (2015). Doing biopolitics differently? Radical potential in the post‐2015 MDG and SDG debates. Globalizations, 12(4), 597–612. 10.1080/14747731.2015.1033172

[wrcr24091-bib-0045] Dell'Angelo, J. , D'Odorico, P. , & Rulli, M. C. (2018). The neglected costs of water peace. Wiley Interdisciplinary Reviews: Water, 5(6), e1316 10.1002/wat2.1316

[wrcr24091-bib-0046] Dermody, B. J. , van Beek, R. P. H. , Meeks, E. , Klein Goldewijk, K. , Scheidel, W. , van der Velde, Y. , Bierkens, M. F. P. , Wassen, M. J. , & Dekker, S. C. (2014). A virtual water network of the Roman world. Hydrology and Earth System Sciences, 18(12), 5025–5040. 10.5194/hess-18-5025-2014

[wrcr24091-bib-0047] Deryugina, T. , & Konar, M. (2017). Impacts of crop insurance on water withdrawals for irrigation. Advances in Water Resources, 110, 437–444. 10.1016/j.advwatres.2017.03.013

[wrcr24091-bib-0048] Di Baldassarre, G. , Kemerink, J. S. , Kooy, M. , & Brandimarte, L. (2014). Floods and societies: The spatial distribution of water‐related disaster risk and its dynamics. WIRES Water, 1(2), 133–139. 10.1002/wat2.1015

[wrcr24091-bib-0049] Di Baldassarre, G. , Kooy, M. , Kemerink, J. S. , & Brandimarte, L. (2013). Towards understanding the dynamic behaviour of floodplains as human‐water systems. Hydrology and Earth System Sciences, 17(8), 3235–3244. 10.5194/hess-17-3235-2013

[wrcr24091-bib-0050] Di Baldassarre, G. , Kreibich, H. , Vorogushyn, S. , Aerts, J. , Arnbjerg‐Nielsen, K. , Barendrecht, M. , Bates, P. , Borga, M. , Botzen, W. , Bubeck, P. , De Marchi, B. , Llasat, C. , Mazzoleni, M. , Molinari, D. , Mondino, E. , Mård, J. , Petrucci, O. , Scolobig, A. , Viglione, A. , & Ward, P. J. (2018). Hess opinions: An interdisciplinary research agenda to explore the unintended consequences of structural flood protection. Hydrology and Earth System Sciences, 22(11), 5629–5637. 10.5194/hess-22-5629-2018

[wrcr24091-bib-0051] Di Baldassarre, G. , Martinez, F. , Kalantari, Z. , & Viglione, A. (2017). Drought and flood in the Anthropocene: Feedback mechanisms in reservoir operation. Earth System Dynamics, 8(1), 225–233. 10.5194/esd-8-225-2017

[wrcr24091-bib-0052] Di Baldassarre, G. , Viglione, A. , Carr, G. , Kuil, L. , Salinas, J. L. , & Blöschl, G. (2013). Sociohydrology: Conceptualising human‐flood interactions. Hydrology and Earth System Sciences, 17(8), 3295–3303. 10.5194/hess-17-3295-2013

[wrcr24091-bib-0053] Di Baldassarre, G. , Viglione, A. , Carr, G. , Kuil, L. , Yan, K. , Brandimarte, L. , & Blöschl, G. (2015). Perspectives on sociohydrology: Capturing feedbacks between physical and social processes. Water Resources Research, 51, 4770–4781. 10.1002/2014WR016416

[wrcr24091-bib-0054] Di Baldassarre, G. , Wanders, N. , AghaKouchak, A. , Kuil, L. , Rangecroft, S. , Veldkamp, T. I. E. , Garcia, M. , van Oel, P. R. , Breinl, K. , & Van Loon, A. F. (2018). Water shortages worsened by reservoir effects. Nature Sustainability, 1(11), 617–622. 10.1038/s41893-018-0159-0

[wrcr24091-bib-0055] Di Baldassarre, G. , Yan, K. , Ferdous, M. D. , & Brandimarte, L. (2014). The interplay between human population dynamics and flooding in Bangladesh: A spatial analysis. Proceedings of the International Association of Hydrological Sciences, 364, 188–191. 10.5194/piahs-364-188-2014

[wrcr24091-bib-0056] Dinda, S. (2004). Environmental Kuznets curve hypothesis: A survey. Ecological Economics, 49(4), 431–455. 10.1016/j.ecolecon.2004.02.011

[wrcr24091-bib-0057] Dodman, D. , Leck, H. , Rusca, M. , & Colenbrander, S. (2017). African urbanisation and urbanism: Implications for risk accumulation and reduction. International Journal of Disaster Risk Reduction, 26, 7–15. 10.1016/j.ijdrr.2017.06.029

[wrcr24091-bib-0058] D'Odorico, P. , Davis, K. F. , Rosa, L. , Carr, J. A. , Chiarelli, D. , Dell'Angelo, J. , Gephart, J. , MacDonald, G. K. , Seekell, D. A. , Suweis, S. , & Rulli, M. C. (2018). The global food‐energy‐water nexus. Reviews of Geophysics, 56, 456–531. 10.1029/2017RG000591

[wrcr24091-bib-0059] Doyle, J. , Francis, B. , & Tannenbaum, A. (1990). Feedback control: Theory and design. Automatica, 22(6), 761–762.

[wrcr24091-bib-0060] Driscoll, D. L. , Appiah‐Yeboah, A. , Salib, P. , & Rupert, D. J. (2007). Merging qualitative and quantitative data in mixed methods research: How to and why not. Ecological and Environmental Anthropology, 3(1), 19–28.

[wrcr24091-bib-0061] Du, E. , Cai, X. , Sun, Z. , & Minsker, B. (2017). Exploring the role of social media and individual behaviors in flood evacuation processes: An agent‐based modeling approach. Water Resources Research, 53, 9164–9180. 10.1002/2017WR021192

[wrcr24091-bib-0062] Dumont, A. , Mayor, B. , & López‐Gunn, E. (2013). Is the rebound effect or Jevons paradox a useful concept for better management of water resources? Insights from the irrigation modernisation process in Spain. Aquatic Procedia, 1, 64–76. 10.1016/j.aqpro.2013.07.006

[wrcr24091-bib-0063] Ekers, M. , & Loftus, A. (2008). The power of water: developing dialogues between Gramsci and Foucault. Environmental Planning D: Society and Space, 26(4), 698–718. 10.1068/d5907

[wrcr24091-bib-0064] Elshafei, Y. , Sivapalan, M. , Tonts, M. , & Hipsey, M. R. (2014). A prototype framework for models of sociohydrology: Identification of key feedback loops with application to two Australian case studies. Hydrology and Earth System Sciences, 18(6), 2141–2166. 10.5194/hess-18-2141-2014

[wrcr24091-bib-0065] Elshafei, Y. , Tonts, M. , Sivapalan, M. , & Hipsey, M. R. (2016). Sensitivity of emergent sociohydrologic dynamics to internal system properties and external sociopolitical factors: Implications for water management. Water Resources Research, 52, 4944–4966. 10.1002/2015WR017944

[wrcr24091-bib-0066] Elster, J. (2007). Explaining social behaviour, (p. 469). Cambridge: Cambridge University Press.

[wrcr24091-bib-0068] Ercumen, A. , Arnold, B. F. , Kumpel, E. , Burt, Z. , Ray, I. , Nelson, K. , & Colford, J. M. Jr. (2015). Upgrading a piped water supply from intermittent to continuous delivery and association with waterborne illness: A matched cohort study in urban India. PLoS medicine, 12(10), e1001892 10.1371/journal.pmed.1001892 26505897 PMC4624240

[wrcr24091-bib-0069] Falkenmark, M. , & Rockström, J. (2008). Building resilience to drought in desertification‐prone savannahs in Sub‐Saharan Africa: The water perspective. Natural Resources Forum, 32(2), 93–102. 10.1111/j.1477-8947.2008.00177.x

[wrcr24091-bib-0070] Fernald, A. , Guldan, S. , Boykin, K. , Cibils, A. , Gonzales, M. , Hurd, B. , Lopez, S. , Ochoa, C. , Ortiz, M. , Rivera, J. , Rodriguez, S. , & Steele, C. (2015). Linked hydrologic and social systems that support resilience of traditional irrigation communities. Hydrology and Earth System Sciences, 19, 293–307. 10.5194/hess-19-293-2015

[wrcr24091-bib-0071] Folke, C. (2006). Resilience: The emergence of a perspective for social–ecological systems analyses. Global Environmental Change, 16(3), 253–267. 10.1016/j.gloenvcha.2006.04.002

[wrcr24091-bib-0072] Folke, C. (2010). How resilient are ecosystems to global environmental change? Sustainability Science, 5(2), 151–154. 10.1007/s11625-010-0109-x

[wrcr24091-bib-0073] Folke, C. , Carpenter, S. R. , Walker, B. , Scheffer, M. , Chapin, T. , & Rockström, J. (2010). Resilience thinking: Integrating resilience, adaptability and transformability. Ecology and Society, 15(4). 10.5751/ES-03610-150420

[wrcr24091-bib-0074] Folke, C. , Hahn, T. , Olsson, P. , & Norberg, J. (2005). Adaptive governance of social‐ecological systems. Annual Review of Environmental Resources, 30(1), 441–473. 10.1146/annurev.energy.30.050504.144511

[wrcr24091-bib-0075] Foster, K. R. , Vecchia, P. , & Repacholi, M. H. (2000). Science and the precautionary principle. Science, 288(5468), 979–981. 10.1126/science.288.5468.979 10841718

[wrcr24091-bib-0076] Frankel, J. A. , & Romer, D. (1999). Does trade cause growth? The American Economic Review, 89(3), 379–399. 10.1257/aer.89.3.379

[wrcr24091-bib-0077] Fukuda‐Parr, S. (2016). From the millennium development goals to the sustainable development goals: Shifts in purpose, concept, and politics of global goal setting for development. Gender and Development, 24(1), 43–52. 10.1080/13552074.2016.1145895

[wrcr24091-bib-0078] Gaál, L. , Szolgay, J. , Kohnová, S. , Parajka, J. , Merz, R. , Viglione, A. , & Blöschl, G. (2012). Flood timescales: Understanding the interplay of climate and catchment processes through comparative hydrology. Water Resources Research, 48, W04511 10.1029/2011WR011509

[wrcr24091-bib-0079] Gal, D. (2018, October 6). Why is behavioral economics so popular? The New York Times. Retrieved from https://www.nytimes.com

[wrcr24091-bib-0080] Garcia, M. , Portney, K. , & Islam, S. (2016). A question driven socio‐hydrological modeling process. Hydrology and Earth System Sciences, 20, 73–92. 10.5194/hess-20-73-2016

[wrcr24091-bib-0081] Geels, F. W. (2005). Processes and patterns in transitions and system innovations: Refining the co‐evolutionary multi‐level perspective. Technological Forecasting and Social Change, 72(6), 681–696. 10.1016/j.techfore.2004.08.014

[wrcr24091-bib-0082] Gilbert N. (Ed) (2008). Researching Social Life, (p. 576). London, UK: Sage Publications.

[wrcr24091-bib-0083] Gilbert, N. , & Terna, P. (2000). How to build and use agent‐based models in social science. Mind & Society, 1(1), 57–72. 10.1007/BF02512229

[wrcr24091-bib-0084] Giuliani, M. , Li, Y. , Castelletti, A. , & Gandolfi, C. (2016). A coupled human‐natural systems analysis of irrigated agriculture under changing climate. Water Resources Research, 52, 6928–6947. 10.1002/2016WR019363

[wrcr24091-bib-0085] Gleick, P. , & Palaniappan, M. (2010). Peak water limits to freshwater withdrawal and use. Proceedings of the National Academy of Sciences of USA, 107(25), 11,155–11,162. 10.1073/pnas.1004812107 PMC2895062 20498082

[wrcr24091-bib-0086] Gleick, P. H. (2014). Water, drought, climate change, and conflict in Syria. Weather, Climate, and Society, 6(3), 331–340. 10.1175/WCAS-D-13-00059.1

[wrcr24091-bib-0087] Global Water Partnership , (2009). A handbook for integrated water resources management in basins. Global Water Partnership (Stockholm, Sweden) and the International Network of Basin Organizations (Madrid, Spain), 104p. ISBN: 978‐91‐85321‐72‐8

[wrcr24091-bib-0089] Gober, P. , & Wheater, H. S. (2015). Debates—Perspectives on sociohydrology: Modeling flood risk as a public policy problem. Water Resources Research, 51, 4782–4788. 10.1002/2015WR016945

[wrcr24091-bib-0090] Gohari, A. , Eslamian, S. , Mirchi, A. , Abedi‐Koupaei, J. , Massah Bavani, A. , & Madani, K. (2013). Water transfer as a solution to water shortage: A fix that can backfire. Journal of Hydrology, 491, 23–39. 10.1016/j.jhydrol.2013.03.021

[wrcr24091-bib-0091] Gonzales, P. , & Ajami, N. (2017). Social and structural patterns of drought‐related water conservation and rebound. Water Resources Research, 53, 10,619–10,634. 10.1002/2017WR021852

[wrcr24091-bib-0092] Grames, J. , Prskawetz, A. , Grass, D. , Viglione, A. , & Blöschl, G. (2016). Modeling the interaction between flooding events and economic growth. Ecological Economics, 129, 193–209. 10.1016/j.ecolecon.2016.06.014

[wrcr24091-bib-0093] Gray, C. L. , & Mueller, V. (2012). Natural disasters and population mobility in Bangladesh. Proceedings of the National Academy of Sciences, 109(16), 6000–6005. 10.1073/pnas.1115944109 PMC3341015 22474361

[wrcr24091-bib-0094] Grey, D. , & Sadoff, C. (2003). Beyond the river: The benefits of cooperation on international rivers. Water Science and Technology, 47(6), 91–96. 10.2166/wst.2003.0365 12731775

[wrcr24091-bib-0095] Griggs, D. , Stafford‐Smith, M. , Gaffney, O. , Rockström, J. , Öhman, M. C. , Shyamsundar, P. , Steffen, W. , Glaser, G. , Kanie, N. , & Noble, I. (2013). Policy: Sustainable Development Goals for people and planet. Nature, 495(7441), 305–307. 10.1038/495305a 23518546

[wrcr24091-bib-0096] Gunda, T. , Turner, B. L. , & Tidwell, V. C. (2018). The influential role of sociocultural feedbacks on community‐managed irrigation system behaviors during times of water stress. Water Resources Research, 54, 2697–2714. 10.1002/2017WR021223

[wrcr24091-bib-0097] Gunderson, L. H. , & Holling, C. S. (2001). Panarchy: Understanding transformations in human and natural systems, (p. 507). Washington DC: Island Press.

[wrcr24091-bib-0098] Haasnoot, M. , Kwakkel, J. H. , Walker, W. E. , & ter Maat, J. (2013). Dynamic adaptive policy pathways: A method for crafting robust decisions for a deeply uncertain world. Global Environmental Change, 23(2), 485–498. 10.1016/j.gloenvcha.2012.12.006

[wrcr24091-bib-0099] Hanna‐Attisha, M. , LaChance, J. , Sadler, R. C. , & Schnepp, A. C. (2016). Elevated blood lead levels in children associated with the Flint drinking water crisis: A spatial analysis of risk and public health response. American Journal of Public Health, 106(2), 283–290. 10.2105/AJPH.2015.303003 26691115 PMC4985856

[wrcr24091-bib-0100] Hoekstra, A. Y. , & Mekonnen, M. M. (2012). The water footprint of humanity. Proceedings of the National Academy of Sciences of USA, 109(9), 3232–3237. 10.1073/pnas.1109936109 PMC3295316 22331890

[wrcr24091-bib-0102] Hulme, M. (2010). Problems with making and governing global kinds of knowledge. Global Environmental Change, 20(4), 558–564. 10.1016/j.gloenvcha.2010.07.005

[wrcr24091-bib-0103] Hussein, H. , Menga, F. , & Greco, F. (2018). Monitoring transboundary water cooperation in SDG 6.5. 2: How a critical hydropolitics approach can spot inequitable outcomes. Sustainability, 10(10), 3640 10.3390/su10103640

[wrcr24091-bib-0104] International Water Management Institute . (2018). Fixing food: Best practices towards the sustainable development goals. International Water Management Institute, 40p.

[wrcr24091-bib-0105] Jevons, W. S. (1866). The coal question (2nd edition), Chapter VII. London, U.K: Macmillan and Co.

[wrcr24091-bib-0106] Jick, T. D. (1979). Mixing qualitative and quantitative methods: Triangulation in action. Administrative Science Quarterly, 24(4), 602–611. 10.2307/2392366

[wrcr24091-bib-0107] Kahneman, D. , & Tversky, A. (1979). Prospect theory: An analysis of decision under risk. Econometrica, 47(2), 263–292. 10.2307/1914185

[wrcr24091-bib-0108] Kallis, G. (2010). Coevolution in water resource development: The vicious cycle of water supply and demand in Athens, Greece. Ecological Economics, 69(4), 796–809. 10.1016/j.ecolecon.2008.07.025

[wrcr24091-bib-0109] Kandasamy, J. , Sounthararajah, D. , Sivabalan, P. , Chanan, A. , Vigneswaran, S. , & Sivapalan, M. (2014). Socio‐hydrologic drivers of the pendulum swing between agriculture development and environmental health: A case study from Murrumbidgee river basin, Australia. Hydrology and Earth System Sciences, 18, 1027–1041. 10.5194/hess-18-1027-2014

[wrcr24091-bib-0110] Karlsson‐Vinkhuyzen, S. , Dahl, A. L. , & Persson, Å. (2018). The emerging accountability regimes for the Sustainable Development Goals and policy integration: Friend or foe? Environment and Planning C: Politics and Space, 36(8), 1371–1390.

[wrcr24091-bib-0111] Kasprzyk, J. R. , Smith, R. M. , Stillwell, A. S. , Madani, K. , Ford, D. , McKinney, D. , & Sorooshian, S. (2018). Defining the role of water resources systems analysis in a changing future. Journal of Water Resources Planning and Management, 144(12), 01818003 10.1061/(ASCE)WR.1943-5452.0001010

[wrcr24091-bib-0112] Kates, R. W. , Colten, C. E. , Laska, S. , & Leatherman, S. P. (2006). Reconstruction of New Orleans after Hurricane Katrina: A research perspective. Proceedings of the National Academy of Sciences of USA, 103(40), 14,653–14,660. 10.1073/pnas.0605726103 PMC1595407 17003119

[wrcr24091-bib-0113] Knight, J. , & Gunatilaka, R. (2010). The rural–urban divide in China: Income but not happiness? The Journal of Development Studies, 46(3), 506–534. 10.1080/00220380903012763

[wrcr24091-bib-0114] Kollock, P. (1998). Social dilemmas: The anatomy of cooperation. Annual Review of Sociology, 24(1), 183–214. 10.1146/annurev.soc.24.1.183

[wrcr24091-bib-0115] Konar, M. , Dalin, C. , Suweis, S. , Hanasaki, N. , Rinaldo, A. , & Rodriguez‐Iturbe, I. (2011). Water for food: The global virtual water trade network. Water Resources Research, 47, W05520 10.1029/2010WR010307 PMC3341016 22474363

[wrcr24091-bib-0116] Konar, M. , Evans, T. P. , Levy, M. , Scott, C. A. , Troy, T. J. , Vörösmarty, C. J. , & Sivapalan, M. (2016). Water resources sustainability in a globalizing world: Who uses the water? Hydrological Processes, 30(18), 3330–3336. 10.1002/hyp.10843

[wrcr24091-bib-0117] Konar, M. , Garcia, M. , Yu, D. J. , Sanderson, M. , & Sivapalan, M. (2018). Expanding the scope and foundation of sociohydrology as the science of coupled human‐water systems. Water Resources Research, 55, 874–887. 10.1029/2018WR024088

[wrcr24091-bib-0118] Kreibich, H. , di Baldassarre, G. , Vorogushyn, S. , Aerts, J. C. J. H. , Apel, H. , Aronica, G. T. , Arnbjerg‐Nielsen, K. , Bouwer, L. M. , Bubeck, P. , Caloiero, T. , Chinh, D. T. , Cortès, M. , Gain, A. K. , Giampá, V. , Kuhlicke, C. , Kundzewicz, Z. W. , Llasat, M. C. , Mård, J. , Matczak, P. , Mazzoleni, M. , Molinari, D. , Dung, N. V. , Petrucci, O. , Schröter, K. , Slager, K. , Thieken, A. H. , Ward, P. J. , & Merz, B. (2017). Adaptation to flood risk: Results of international paired flood event studies. Earth's Future, 5(10), 953–965. 10.1002/2017EF000606

[wrcr24091-bib-0119] Kuil, L. , Carr, G. , Viglione, A. , Prskawetz, A. , & Blöschl, G. (2016). Conceptualizing socio‐hydrological drought processes: The case of the Maya collapse. Water Resources Research, 52, 6222–6242. 10.1002/2015WR018298 27840455 PMC5091644

[wrcr24091-bib-0120] Kuil, L. , Evans, T. , McCord, P. F. , Salinas, J. L. , & Blöschl, G. (2018). Exploring the influence of smallholders' perceptions regarding water availability on crop choice and water allocation through socio‐hydrological modeling. Water Resources Research, 54, 2580–2604. 10.1002/2017WR021420 PMC6559291 31217644

[wrcr24091-bib-0121] Kuil, L. , Prskawetz, A. , Blöschl, G. , Viglione, A. , Carr, G. , & Salinas, J. L. (2019). Learning from the Ancient Maya: Exploring the impact of drought on population dynamics. Ecological Economics, 157, 1–16. 10.1016/j.ecolecon.2018.10.018

[wrcr24091-bib-0122] Kumpel, E. , & Nelson, K. L. (2013). Comparing microbial water quality in an intermittent and continuous piped water supply. Water research, 47(14), 5176–5188. 10.1016/j.watres.2013.05.058 23866140

[wrcr24091-bib-0123] Kumpel, E. , & Nelson, K. L. (2016). Intermittent water supply: Prevalence, practice, and microbial water quality. Environmental science & technology, 50(2), 542–553. 10.1021/acs.est.5b03973 26670120

[wrcr24091-bib-0124] Langford, M. (2016). Lost in transformation? The politics of the sustainable development goals. Ethics & International Affairs, 30(2), 167–176. 10.1017/S0892679416000058

[wrcr24091-bib-0125] Lant, C. , Baggio, J. , Konar, M. , Meija, A. , Ruddell, B. , Rushforth, R. , Sabo, J. , & Troy, T. (2018). The U.S. food‐energy‐water system: A blueprint to fill the mesoscale gap for science and decision‐making. Ambio, 48(3), 251–263. 10.1007/s13280-018-1077-0 29981010 PMC6374226

[wrcr24091-bib-0126] Leong, C. (2018). The role of narratives in sociohydrological models of flood behaviors. Water Resources Research, 54, 3100–3121. 10.1002/2017WR022036

[wrcr24091-bib-0127] Liu, D. , Tian, F. , Lin, M. , & Sivapalan, M. (2015). A conceptual socio‐hydrological model of the co‐evolution of humans and water: Case study of the Tarim River basin, western China. Hydrology and Earth System Sciences, 19, 1035–1054. 10.5194/hess-19-1035-2015

[wrcr24091-bib-0128] Liu, G. , Zhang, Y. , Knibbe, W. J. , Feng, C. , Liu, W. , Medema, G. , & van der Meer, W. (2017). Potential impacts of changing supply‐water quality on drinking water distribution: A review. Water Research, 116, 135–148. 10.1016/j.watres.2017.03.031 28329709

[wrcr24091-bib-0129] Liu, J. , Dietz, T. , Carpenter, S. R. , Alberti, M. , Folke, C. , Moran, E. , Pell, A. N. , Deadman, P. , Kratz, T. , Lubchenco, J. , Ostrom, E. , Ouyang, Z. , Provencher, W. , Redman, C. L. , Schneider, S. H. , & Taylor, W. W. (2007). Complexity of coupled human and natural systems. Science, 317(5844), 1513–1516. 10.1126/science.1144004 17872436

[wrcr24091-bib-0130] Liu, J. , Yang, H. , Cudennec, C. , Gain, A. K. , Hoff, H. , Lawford, R. , Qi, J. , de Strasser, L. , Yillia, P. T. , & Zheng, C. (2017). Challenges in operationalizing the water‐energy‐food nexus. Hydrological Sciences Journal, 62(11), 1714–1720. 10.1080/02626667.2017.1353695

[wrcr24091-bib-0131] Liu, Y. (2016). Co‐evolution and driving mechanism of coupled socio‐hydrological system in arid areas. PhD dissertation. Beijing, China: Department of Hydraulic Engineering, Tsinghua University.

[wrcr24091-bib-0132] Liu, Y. , Tian, F. , Hu, H. , & Sivapalan, M. (2013). Socio‐hydrologic perspectives of the co‐evolution of humans and water in the Tarim River Basin, Western China: The Taiji–Tire Model. Hydrology Earth System Sciences, 18, 1289–1303.

[wrcr24091-bib-0133] Liverman, D. M. (2018). Geographic perspectives on development goals: Constructive engagements and critical perspectives on the MDGs and the SDGs. Dialogues in Human Geography, 8(2), 168–185. 10.1177/2043820618780787

[wrcr24091-bib-0134] Loucks, D. P. (2015). Debates—Perspectives on sociohydrology: Simulating hydrologic‐human interactions. Water Resources Research, 51, 4789–4794. 10.1002/2015WR017002

[wrcr24091-bib-0135] Loucks, D. P. , Stedinger, J. R. , Davis, D. W. , & Stakhiv, E. Z. (2008). Private and public responses to flood risks. International Journal of Water Resources Development, 24(4), 537–549.

[wrcr24091-bib-0136] Loucks, D. P. , Stedinger, J. R. , & Stakhiv, E. Z. (2006). Individual and societal responses to natural hazards. Journal of Water Resources Planning and Management, 132(5), 315–319. 10.1061/(ASCE)0733-9496(2006)132:5(315)

[wrcr24091-bib-0137] Loucks, D. P. , Van Beek, E. , Stedinger, J. R. , Dijkman, J. P. , & Villars, M. T. (2005). Water resources systems planning and management: An introduction to methods, models and applications. 680 pages. Paris: UNESCO.

[wrcr24091-bib-0138] Ludy, J. , & Kondolf, G. M. (2012). Flood risk perception in lands “protected” by 100‐year levees. Natural Hazards, 61(2), 829–842. 10.1007/s11069-011-0072-6

[wrcr24091-bib-0139] Mabogunje, A. L. (1970). Systems approach to a theory of rural‐urban migration. Geographical analysis, 2(1), 1–18. 10.1111/j.1538-4632.1970.tb00140.x

[wrcr24091-bib-0140] Madani, K. , & Dinar, A. (2012). Non‐cooperative institutions for sustainable common pool resource management: Application to groundwater. Ecological Economics, 74, 34–45. 10.1016/j.ecolecon.2011.12.006

[wrcr24091-bib-0141] Mahoney, J. (2000). Path dependence in historical sociology. Theory and Society, 29(4), 507–548. 10.1023/A:1007113830879

[wrcr24091-bib-0142] Mallakpour, I. , AghaKouchak, A. , & Sadegh, M. (2019). Climate‐induced changes in the risk of hydrological failure of major dams in California. Geophysical Research Letters, 46, 2130–2139. 10.1029/2018GL081888

[wrcr24091-bib-0143] Marshall, A. (1890). Principles of economics, (First ed.). London 731: Macmillan.

[wrcr24091-bib-0144] Marston, L. , Ao, Y. , Konar, M. , Mekonnen, M. , & Hoekstra, A. Y. (2018). High‐resolution water footprints of production of the United States. Water Resources Research, 54, 2288–2316. 10.1002/2017WR021923

[wrcr24091-bib-0145] Marston, L. , & Konar, M. (2017). Drought impacts to water footprints and virtual water transfers of the Central Valley of California. Water Resources Research, 53, 5756–5773. 10.1002/2016WR020251

[wrcr24091-bib-0146] Masozera, M. , Bailey, M. , & Kerchner, C. (2007). Distribution of impacts of natural disasters across income groups: A case study of New Orleans. Ecological Economics, 63(2‐3), 299–306. 10.1016/j.ecolecon.2006.06.013

[wrcr24091-bib-0147] Massey, D. S. , Arango, J. , Hugo, G. , Kouaouci, A. , Pellegrino, A. , & Taylor, J. E. (1993). Theories of international migration: A review and appraisal. Population and development review, 19(3), 431–466. 10.2307/2938462

[wrcr24091-bib-0148] Massuel, S. , Riaux, J. , Molle, F. , Kuper, M. , Ogilvie, A. , Collard, A. L. , Leduc, C. , & Barreteau, O. (2018). Inspiring a broader socio‐hydrological negotiation approach with interdisciplinary field‐based experience. Water Resources Research, 54, 2510–2522. 10.1002/2017WR021691

[wrcr24091-bib-0149] Mawdsley, E. (2017). Development geography 1: Cooperation, competition and convergence between ‘north’ and ‘south’. Progress in Human Geography, 41(1), 108–117. 10.1177/0309132515601776

[wrcr24091-bib-0150] Mawdsley, E. (2018). From billions to trillions Financing the SDGs in a world beyond aid. Dialogues in Human Geography, 8(2), 191–195. 10.1177/2043820618780789

[wrcr24091-bib-0151] McMichael, P. (2017). The shared humanity of global development: Bio‐politics and the SDGs. Globalizations, 14(3), 335–336. 10.1080/14747731.2017.1281627

[wrcr24091-bib-0152] McMillan, H. , Montanari, A. , Cudennec, C. , Savenije, H. , Kreibich, H. , Krueger, T. , Liu, J. , Mejia, A. , van Loon, A. , Aksoy, H. , di Baldassarre, G. , Huang, Y. , Mazvimavi, D. , Rogger, M. , Sivakumar, B. , Bibikova, T. , Castellarin, A. , Chen, Y. , Finger, D. , Gelfan, A. , Hannah, D. M. , Hoekstra, A. Y. , Li, H. , Maskey, S. , Mathevet, T. , Mijic, A. , Pedrozo Acuña, A. , Polo, M. J. , Rosales, V. , Smith, P. , Viglione, A. , Srinivasan, V. , Toth, E. , van Nooyen, R. , & Xia, J. (2016). Panta Rhei 2013–2015: Global perspectives on hydrology, society and change. Hydrological Sciences Journal, 61(7), 1–18. 10.1080/02626667.2016.1159308

[wrcr24091-bib-0153] Mechler, R. , & Bouwer, L. M. (2015). Understanding trends and projections of disaster losses and climate change: Is vulnerability the missing link? Climatic Change, 133(1), 23–35. 10.1007/s10584-014-1141-0

[wrcr24091-bib-0154] Merme, V. , Ahlers, R. , & Gupta, J. (2014). Private equity, public affair: Hydropower financing in the Mekong basin. Global Environmental Change, 24, 20–29. 10.1016/j.gloenvcha.2013.11.007

[wrcr24091-bib-0155] Mianabadi, M. , Mostert, E. , Pande, S. , & van de Giesen, N. (2015). Weighted bankruptcy rules and transboundary water resources allocation. Water Resources Management, 29, 2303–2321. 10.1007/s11269-015-0942-x

[wrcr24091-bib-0156] Michael, H. A. , & Voss, C. I. (2008). Evaluation of the sustainability of deep groundwater as an arsenic‐safe resource in the Bengal basin. Proceedings of the National Academy of Sciences, 105(25), 8531–8536. 10.1073/pnas.0710477105 PMC2438411 18562284

[wrcr24091-bib-0157] Milly, P. C. D. , Betancourt, J. , Falkenmark, M. , Hirsch, R. M. , Kundzewicz, Z. W. , Lettenmaier, D. P. , & Stouffer, R. J. (2008). Stationarity is dead: Whither water management? Science, 319, 573–574. 10.1126/science.1151915 18239110

[wrcr24091-bib-0158] Mitchell, M. (2009). Complexity: A guided tour. Oxford: Oxford University Press.

[wrcr24091-bib-0159] Molle, F. , Mollinga, P. P. , & Wester, P. (2009). Hydraulic bureaucracies and the hydraulic mission: Flows of water, flows of power. Water Alternatives, 2(3), 328–349.

[wrcr24091-bib-0160] Montanari, A. , Young, G. , Savenije, H. H. G. , Hughes, D. , Wagener, T. , Ren, L. L. , Koutsoyiannis, D. , Cudennec, C. , Toth, E. , Grimaldi, S. , Blöschl, G. , Sivapalan, M. , Beven, K. , Gupta, H. , Hipsey, M. , Schaefli, B. , Arheimer, B. , Boegh, E. , Schymanski, S. J. , di Baldassarre, G. , Yu, B. , Hubert, P. , Huang, Y. , Schumann, A. , Post, D. A. , Srinivasan, V. , Harman, C. , Thompson, S. , Rogger, M. , Viglione, A. , McMillan, H. , Characklis, G. , Pang, Z. , & Belyaev, V. (2013). "Panta Rhei—Everything flows": Change in hydrology and society—The IAHS Scientific Decade 2013–2022. Hydrological Sciences Journal, 58(6), 1256–1275. 10.1080/02626667.2013.809088

[wrcr24091-bib-0161] Morrow, J. D. (1994). Game theory for political scientists. No. 30, 519.83. Princeton, NJ: Princeton University Press.

[wrcr24091-bib-0162] Morton, S. , Pencheon, D. , & Squires, N. (2017). Sustainable Development Goals (SDGs), and their implementation: A national global framework for health, development and equity needs a systems approach at every level. British Medical Bulletin, 124(1), 81–90. 10.1093/bmb/ldx031 29069332

[wrcr24091-bib-0163] Mostert, E. (2018). An alternative approach for sociohydrology: Case study research. Hydrological Earth System Sciences, 22(1), 317–329. 10.5194/hess-22-317-2018

[wrcr24091-bib-0164] Müller, M. F. , & Levy, M. (2019). Complementary vantage points: Integrating hydrology and economics for sociohydrologic knowledge generation. Water Resources Research, 55, 2549–2571. 10.1029/2019WR024786

[wrcr24091-bib-0165] Müller, M. F. , Müller‐Itten, M. C. , & Gorelick, S. M. (2017). How Jordan and Saudi Arabia are avoiding a tragedy of the commons over shared groundwater. Water Resources Research, 53, 5451–5468. 10.1002/2016WR020261

[wrcr24091-bib-0166] Müller, M. F. , Yoon, J. , Gorelick, S. M. , Avisse, N. , & Tilmant, A. (2016). Impact of the Syrian refugee crisis on land use and transboundary freshwater resources. Proceedings of the national academy of sciences, 113(52), 14,932–14,937. 10.1073/pnas.1614342113 PMC5206523 27930317

[wrcr24091-bib-0167] Muneepeerakul, R. , & Anderies, J. M. (2017). Strategic behaviors and governance challenges in social‐ecological systems. Earth's Future, 5(8), 865–876. 10.1002/2017EF000562

[wrcr24091-bib-0168] Neumann, J. V. , & Morgenstern, O. (1944). Theory of games and economic behavior, (p. 625). Princeton, NJ: Princeton University Press.

[wrcr24091-bib-0169] Nilsson, M. , Griggs, D. , & Visbeck, M. (2016). Map the interactions between Sustainable Development Goals. Nature, 534(7607), 320–322. 10.1038/534320a 27306173

[wrcr24091-bib-0170] Noël, P. H. , & Cai, X. (2017). On the role of individuals in models of coupled human and natural systems: Lessons from a case study in the Republican River basin. Environmental Modelling & Software, 92, 1–16. 10.1016/j.envsoft.2017.02.010

[wrcr24091-bib-0171] Null, C. , Stewart, C. P. , Pickering, A. J. , Dentz, H. N. , Arnold, B. F. , Arnold, C. D. , Benjamin‐Chung, J. , Clasen, T. , Dewey, K. G. , Fernald, L. C. H. , Hubbard, A. E. , Kariger, P. , Lin, A. , Luby, S. P. , Mertens, A. , Njenga, S. M. , Nyambane, G. , Ram, P. K. , & Colford, J. M. Jr. (2018). Effects of water quality, sanitation, handwashing, and nutritional interventions on diarrhoea and child growth in rural Kenya: A cluster‐randomised controlled trial. Lancet Global Health, 6(3), e316–e329. 10.1016/S2214-109X(18)30005-6 29396219 PMC5809717

[wrcr24091-bib-0172] Olson, M. (1965). The logic of collective action: Public goods and the theory of groups. Cambridge, MA: Harvard University Press.

[wrcr24091-bib-0173] Ostrom, E. (1990). Governing the commons: The evolution of institutions for collective action, (p. 271). Cambridge, Massachusetts: Cambridge University Press.

[wrcr24091-bib-0174] Ostrom, E. (2009). A general framework for analysing sustainability of social‐ecological systems. Science, 325(5939), 419–422. 10.1126/science.1172133 19628857

[wrcr24091-bib-0175] Pahl‐Wostl, C. , Becker, G. , Knieper, C. , & Sendzimir, J. (2013). How multilevel societal learning processes facilitate transformative change: A comparative case study analysis on flood management. Ecology and Society, 18(4), 58.

[wrcr24091-bib-0176] Pande, S. , & Ertsen, M. (2014). Endogenous change: on cooperation and water in ancient history. Hydrology and Earth System Sciences, 18(5), 1745–1760. 10.5194/hess-18-1745-2014

[wrcr24091-bib-0177] Pande, S. , Ertsen, M. , & Sivapalan, M. (2014). Endogenous technological and population change under increasing water scarcity. Hydrology and Earth System Sciences, 18(8), 3239–3258. 10.5194/hess-18-3239-2014

[wrcr24091-bib-0178] Pande, S. , & Savenije, H. H. G. (2016). A sociohydrological model for smallholder farmers in Maharashtra. India, Water Resources Research, 52, 1923–1947. 10.1002/2015WR017841

[wrcr24091-bib-0179] Pande, S. , & Sivapalan, M. (2017). Progress in sociohydrology: A meta‐analysis of challenges and opportunities. WIRES Water, 4(4), 4, e1193 10.1002/wat2.1193

[wrcr24091-bib-0180] Pande, S. , van Den Boom, B. , Savenije, H. H. G. , & Gosain, A. K. (2011). Water valuation at basin scale with application to western India. Ecological Economics, 70, 2416–2428.

[wrcr24091-bib-0181] Pepper, M. , Roche, C. P. , & Mudd, G. M. (2014). Mining legacies—Understanding life‐of‐mine across time and space In Proceedings of the Life‐of‐Mine Conference (pp. 449–465). Brisbane, Australia: Australasian Institute of Mining and Metallurgy.

[wrcr24091-bib-0182] Petersen‐Perlman, J. D. , Veilleux, J. C. , & Wolf, A. T. (2017). International water conflict and cooperation: Challenges and opportunities. Water International, 42(2), 105–120. 10.1080/02508060.2017.1276041

[wrcr24091-bib-0183] Petersen‐Perlman, J. D. , & Wolf, A. T. (2015). Getting to the first handshake: Enhancing security by initiating cooperation in transboundary river basins. Journal of the American Water Resources Association, 51(6), 1688–1707. 10.1111/1752-1688.12348

[wrcr24091-bib-0184] Pringle, C. (2003). What is hydrologic connectivity and why is it ecologically important? Hydrological Processes, 17(13), 2685–2689. 10.1002/hyp.5145

[wrcr24091-bib-0185] Robinson, J. (2016). Thinking cities through elsewhere: Comparative tactics for a more global urban studies. Progress in Human Geography, 40(1), 3–29. 10.1177/0309132515598025

[wrcr24091-bib-0186] Roby, N. A. , Gonzales, P. , Quesnel, K. J. , & Ajami, N. K. (2018). A novel search algorithm for quantifying news media coverage as a measure of environmental issue salience. Environmental Modelling & Software, 101, 249–255. 10.1016/j.envsoft.2017.12.012

[wrcr24091-bib-0187] Rockström, J. , Steffen, W. , Noone, K. , Persson, Å. , Chapin, F. S. , Lambin, E. F. , Lenton, T. M. , Scheffer, M. , Folke, C. , Schellnhuber, H. J. , Nykvist, B. , de Wit, C. A. , Hughes, T. , van der Leeuw, S. , Rodhe, H. , Sörlin, S. , Snyder, P. K. , Costanza, R. , Svedin, U. , Falkenmark, M. , Karlberg, L. , Corell, R. W. , Fabry, V. J. , Hansen, J. , Walker, B. , Liverman, D. , Richardson, K. , Crutzen, P. , & Foley, J. A. (2009). A safe operating space for humanity. Nature, 461(7263), 472–475. 10.1038/461472a 19779433

[wrcr24091-bib-0188] Rogers, R. W. (1975). A protection motivation theory of fear appeals and attitude change. The Journal of Psychology, 91(1), 93–114. 10.1080/00223980.1975.9915803 28136248

[wrcr24091-bib-0189] Romagny, B. , & Cudennec, C. (2006). Gestion de l'eau en milieu aride: Considérations physiques et sociales pour l'identification des territoires pertinents dans le Sud‐Est tunisien. Développement durable et territoires. Économie, géographie, politique, droit, sociologie, (Dossier 6).

[wrcr24091-bib-0190] Roobavannan, M. , Kandasamy, J. , Pande, S. , Vigneswaran, S. , & Sivapalan, M. (2017). Role of sectoral transformation in the evolution of water management norms in agricultural catchments: A sociohydrologic modeling analysis. Water Resources Research, 53, 8344–8365. 10.1002/2017WR020671

[wrcr24091-bib-0191] Roobavannan, M. , van Emmerik, T. , Elshafei, Y. , Kandasamy, J. , Sanderson, M. , Vigneswaran, S. , Pande, S. , & Sivapalan, M. (2018). Norms and values in socio‐hydrological models. Hydrology and Earth System Sciences, 22, 1337–1349. 10.5194/hess-22-1337-2018

[wrcr24091-bib-0192] Rusca, M. , Alda‐Vidal, C. , Hordijk, M. , & Kral, N. (2017). Bathing without water, and other stories of everyday hygiene practices and risk perception in urban low‐income areas: The case of Lilongwe, Malawi. Environment and Urbanisation, 29(2), 533–550. 10.1177/0956247817700291

[wrcr24091-bib-0194] Rusca, M. , Boakye‐Ansah, A. S. , Loftus, A. , Ferrero, G. , & van der Zaag, P. (2017). An interdisciplinary political ecology of drinking water quality. Exploring socio‐ecological inequalities in Lilongwe's water supply network. Geoforum, 84, 138–146. 10.1016/j.geoforum.2017.06.013

[wrcr24091-bib-0195] Rusca, M. , Schwartz, K. , Hadzovic, L. , & Ahlers, R. (2015). Adapting generic models through bricolage: Elite capture of water users associations in Peri‐urban Lilongwe. The European Journal of Development Research, 27(5), 777–792. 10.1057/ejdr.2014.58

[wrcr24091-bib-0196] Russell, M. (2000). Reducing the nuclear legacy burden: DOE environmental management strategy and implementation. Report Number: JIEE/2000‐01. Joint Institute for Energy and Environment, Knoxville. http://isse.utk.edu/pdf/jieepubs/00‐01.pdf

[wrcr24091-bib-0197] Sanderson, M. R. , Bergtold, J. S. , Heier Stamm, J. L. , Caldas, M. M. , & Ramsey, S. M. (2017). Bringing the “social” into sociohydrology: Conservation policy support in the Central Great Plains of Kansas, USA. Water Resources Research, 53, 6725–6743. 10.1002/2017WR020659

[wrcr24091-bib-0198] Satterthwaite, D. (2016a). Missing the Millennium Development Goal targets for water and sanitation in urban areas. Environment and Urbanization, 28(1), 99–118. 10.1177/0956247816628435

[wrcr24091-bib-0199] Satterthwaite, D. (2016b). Successful, safe and sustainable cities: Towards a new urban agenda. Commonwealth Journal of Local Governance, 19, 3–18.

[wrcr24091-bib-0200] Savenije, H. H. G. , Hoekstra, A. Y. , & van der Zaag, P. (2014). Evolving water science in the Anthropocene. Hydrology and Earth System Sciences, 18(1), 319–332. 10.5194/hess-18-319-2014

[wrcr24091-bib-0201] Savenije, H. H. G. , & Van der Zaag, P. (2008). Integrated water resources management: Concepts and issues. Physics and Chemistry of the Earth, Parts A/B/C, 33(5), 290–297. 10.1016/j.pce.2008.02.003

[wrcr24091-bib-0202] Savio, D. , Sinclair, L. , Ijaz, U. Z. , Parajka, J. , Reischer, G. H. , Stadler, P. , Blaschke, A. P. , Blöschl, G. , Mach, R. L. , Kirschner, A. K. T. , Farnleitner, A. H. , & Eiler, A. (2015). Bacterial diversity along a 2600 km river continuum. Environmental Microbiology, 17(12), 4994–5007. 10.1111/1462-2920.12886 25922985 PMC4918796

[wrcr24091-bib-0203] Schleicher, J. , Schaafsma, M. , & Vira, B. (2018). Will the Sustainable Development Goals address the links between poverty and the natural environment? Current Opinion in Environmental Sustainability, 34, 43–47. 10.1016/j.cosust.2018.09.004

[wrcr24091-bib-0204] Scott, C. A. (2011). The water‐energy‐climate nexus: Resources and policy outlook for aquifers in Mexico. Water Resources Research, 47, W00L04 10.1029/2011WR010805

[wrcr24091-bib-0205] Scott, C. A. , Vicuña, S. , Blanco‐Gutiérrez, I. , Meza, F. , & Varela‐Ortega, C. (2013). Irrigation efficiency and water‐policy implications for river‐basin resilience. Hydrology Earth System Sciences, 18, 1339–1348.

[wrcr24091-bib-0206] Sexsmith, K. , & McMichael, P. (2015). Formulating the SDGs: Reproducing or reimagining state‐centered development? Globalizations, 12(4), 581–596. 10.1080/14747731.2015.1038096

[wrcr24091-bib-0207] Shah, T. , Giordano, M. , & Mukherji, A. (2012). Political economy of the energy‐groundwater nexus in India: Exploring issues and assessing policy options. Hydrogeology Journal, 20(5), 995–1006. 10.1007/s10040-011-0816-0

[wrcr24091-bib-0208] Shaheed, A. , Orgill, J. , Montgomery, M. A. , Jeuland, M. A. , & Brown, J. (2014). Why ‘improved’ water sources are not always safe. Bulletin of the World Health Organization, 92(4), 283–289. 10.2471/BLT.13.119594 24700996 PMC3967570

[wrcr24091-bib-0209] Sivapalan, M. , & Blöschl, G. (2015). Time scale interactions and the coevolution of humans and water. Water Resources Research, 51, 6988–7022. 10.1002/2015WR017896

[wrcr24091-bib-0210] Sivapalan, M. , Konar, M. , Srinivasan, V. , Chhatre, A. , Wutich, A. , Scott, C. A. , Wescoat, J. L. , & Rodriguez‐Iturbe, I. (2014). Sociohydrology: Use‐inspired water sustainability science for the Anthropocene. Earth's Future, 2, 225–230. 10.1002/2013EF000164

[wrcr24091-bib-0211] Sivapalan, M. , Savenjie, H. H. G. , & Blöschl, G. (2012). Sociohydrology: A new science of people and water. Hydrological Processes, 26(8), 1270–1276. 10.1002/hyp.8426

[wrcr24091-bib-0212] Song, J. , & Whittington, D. (2004). Why have some countries on international rivers been successful in negotiating treaties? A global perspective. Water Resources Research, 40, W05S06 10.1029/2003WR002536

[wrcr24091-bib-0213] Srinivasan, V. (2015). Reimagining the past —Use of counterfactual trajectories in socio‐hydrological modelling: The case of Chennai, India. Hydrology and Earth System Sciences, 19, 785–801. 10.5194/hess-19-785-2015

[wrcr24091-bib-0214] Srinivasan, V. , Konar, M. , & Sivapalan, M. (2017). A dynamic framework for water security. Water Security, 1, 12–20. 10.1016/j.wasec.2017.03.001

[wrcr24091-bib-0215] Srinivasan, V. , Lambin, E. F. , Gorelick, S. M. , Thompson, B. H. , & Rozelle, S. (2012). The nature and causes of the global water crisis: Syndromes from a meta‐analysis of coupled human‐water studies. Water Resources Research, 48, W10516 10.1029/2011WR011087

[wrcr24091-bib-0216] Stuart, E. , & Woodroffe, J. (2016). Leaving no‐one behind: Can the Sustainable Development Goals succeed where the Millennium Development Goals lacked? Gender and Development, 24(1), 69–81. 10.1080/13552074.2016.1142206

[wrcr24091-bib-0217] Sultana, F. (2006). Gendered waters, poisoned wells: Political ecology of the arsenic crisis in Bangladesh In Lahiri‐Dutt K. (Ed.), Fluid bonds: Views on gender and water, (pp. 362–386). Canberra: Stree Publishers, India, with Australian National University.

[wrcr24091-bib-0218] Sultana, F. (2011). Suffering for water, suffering from water: Emotional geographies of resource access, control and conflict. Geoforum, 42(2), 163–172. 10.1016/j.geoforum.2010.12.002

[wrcr24091-bib-0219] Sultana, F. (2018). An (Other) geographical critique of development and SDGs. Dialogues in Human Geography, 8(2), 186–190. 10.1177/2043820618780788

[wrcr24091-bib-0220] Swyngedouw, E. (1997). Power, nature, and the city. The conquest of water and the political ecology of urbanization in Guayaquil, Ecuador: 1880–1990. Environment and Planning A, 29(2), 311–332. 10.1068/a290311

[wrcr24091-bib-0221] Ternes, T. , Joss, A. , & Oehlmann, J. (2015). Occurrence, fate, removal and assessment of emerging contaminants in water in the water cycle (from wastewater to drinking water). Water Research, 72, 1–2. 10.1016/j.watres.2015.02.055 25857678

[wrcr24091-bib-0222] Tiwale, S. , Rusca, M. , & Zwarteveen, M. (2018). The power of pipes: Mapping urban water inequities through the material properties of networked water infrastructures. The case of Lilongwe, Malawi. Water Alternatives, 11(2), 314–335.

[wrcr24091-bib-0223] Todaro, M. P. (1969). A model of labor migration and urban unemployment in less developed countries. The American Economic Review, 59(1), 138–148.

[wrcr24091-bib-0224] Tosi Robinson, D. , Schertenleib, A. , Kunwar, B. , Shrestha, R. , Bhatta, M. , & Marks, S. (2018). Assessing the impact of a risk‐based intervention on piped water quality in rural communities: The case of mid‐western Nepal. International journal of environmental research and public health, 15(8), 1616 10.3390/ijerph15081616 PMC6121632 30065180

[wrcr24091-bib-0225] Treuer, G. , Koebele, E. , Deslatte, A. , Ernst, K. , Garcia, M. , & Manago, K. (2017). A narrative method for analyzing transitions in urban water management: The case of the Miami‐Dade Water and Sewer Department. Water Resources Research, 53, 891–908. 10.1002/2016WR019658

[wrcr24091-bib-0226] Troy, T. J. , Konar, M. , Srinivasan, V. , & Thompson, S. (2015). Moving sociohydrology forward: A synthesis across studies. Hydrology and Earth System Sciences, 19(8), 3667–3679. 10.5194/hess-19-3667-2015

[wrcr24091-bib-0227] Troy, T. J. , Pavao‐Zuckerman, M. , & Evans, T. P. (2015). Debates—Perspectives on sociohydrology: Socio‐hydrologic modeling: Tradeoffs, hypothesis testing, and validation. Water Resources Research, 51, 4806–4814. 10.1002/2015WR017046

[wrcr24091-bib-0228] Truelove, Y. (2011). (Re‐)conceptualizing water inequality in Delhi, India through a feminist political ecology framework. Geoforum, 42(2), 143–152. 10.1016/j.geoforum.2011.01.004

[wrcr24091-bib-0229] Truelove, Y. (2016). Incongruent waterworlds: Situating the everyday practices and power of water in Delhi. South Asia Multidisciplinary Academic Journal, 14(14). 10.4000/samaj.4164

[wrcr24091-bib-0230] Turner, B. , Tidwell, V. , Fernald, A. , Rivera, J. , Rodriguez, S. , Guldan, S. , Ochoa, C. , Hurd, B. , Boykin, K. , & Cibils, A. (2016). Modeling acequia irrigation systems using system dynamics: Model development, evaluation, and sensitivity analyses to investigate effects of socio‐economic and biophysical feedbacks. Sustainability, 8(10), 1019 10.3390/su8101019

[wrcr24091-bib-0231] UN Economic and Social Council . 2016 Mainstreaming of the three dimensions of sustainable development throughout the United Nations system. http://www.un.org/ga/search/view_doc.asp?symbol = A/71/76&Lang = E

[wrcr24091-bib-0232] UN Water (2016). Water and sanitation interlinkages across the 2030 agenda for sustainable development. http://www.unwater.org/publications/water‐sanitation‐interlinkages‐across‐2030‐agenda‐sustainable‐development/

[wrcr24091-bib-0233] UN Water (2018). Sustainable Development Goal 6, Synthesis Report 2018 on water and sanitation. Executive Summary, http://www.unwater.org/publications/executive‐summary‐sdg‐6‐synthesis‐report‐2018‐on‐water‐and‐sanitation

[wrcr24091-bib-0234] UN Water (2019). World Water Development Report https://unesdoc.unesco.org/ark:/48223/pf0000367306

[wrcr24091-bib-0236] United Nations Environment Program, UNEP (2016). A snapshot of the world's water quality: Towards a global assessment. In: https://uneplive.unep.org/media/docs/assessments/unep_wwqa_report_web.pdf

[wrcr24091-bib-0237] United Nations Office of Disaster Risk Reduction, UNISDR (2018). UNISDR Annual Report 2017, United Nations Office of Disaster Risk Reduction. United Nations, Geneva. 64p.

[wrcr24091-bib-0238] United Nations, UN (2013). A new global partnership: Eradicate poverty and transform economies through sustainable development. The report of the high‐level panel of eminent persons on the post‐2015 development agenda. United Nations, New York.

[wrcr24091-bib-0239] United Nations, UN (2015). Transforming our world: The 2030 agenda for sustainable development. Resolution adopted by the General Assembly.

[wrcr24091-bib-0241] Van der Ent, R. J. , Savenije, H. H. G. , Schaefli, B. , & Steele‐Dunne, S. C. (2010). Origin and fate of atmospheric moisture over continents. Water Resources Research, 46, W09525 10.1029/2010WR009127

[wrcr24091-bib-0242] Van Emmerik, T. H. M. , Li, Z. , Sivapalan, M. , Pande, S. , Kandasamy, J. , Savenije, H. H. G. , Chanan, A. , & Vigneswaran, S. (2014). Socio‐hydrologic modeling to understand and mediate the competition for water between agriculture development and environmental health: Murrumbidgee River Basin, Australia. Hydrology and Earth System Sciences, 18(10), 4239–4259. 10.5194/hess-18-4239-2014

[wrcr24091-bib-0243] Van Laerhoven, F. , & Ostrom, E. (2007). Traditions and trends in the study of the commons. International Journal of the Commons, 1(1), 3–28. 10.18352/ijc.76

[wrcr24091-bib-0244] Vandemoortele, J. (2011). If not the Millennium Development Goals, then what? Third World Quarterly, 32(1), 9–25. 10.1080/01436597.2011.543809

[wrcr24091-bib-0245] Vandewalle, E. , & Jepson, W. (2015). Mediating water governance: Point‐of‐use water filtration devices for low‐income communities along the US‐Mexico border. Geo: Geography and Environment, 2(2), 107–121.

[wrcr24091-bib-0246] Viglione, A. , Di Baldassarre, G. , Brandimarte, L. , Kuil, L. , Carr, G. , Salinas, J. L. , Scolobig, A. , & Blöschl, G. (2014). Insights from sociohydrology modelling on dealing with flood risk–roles of collective memory, risk‐taking attitude and trust. Journal of Hydrology, 518, 71–82. 10.1016/j.jhydrol.2014.01.018

[wrcr24091-bib-0247] Vörösmarty, C. J. , Pahl‐Wostl, C. , Bunn, S. E. , & Lawford, R. (2013). Global water, the Anthropocene and the transformation of a science. Current Opinion in Environmental Sustainability, 5(6), 539–550. 10.1016/j.cosust.2013.10.005

[wrcr24091-bib-0248] Wagener, T. , Sivapalan, M. , Troch, P. A. , McGlynn, B. L. , Harman, C. J. , Gupta, H. V. , Kumar, P. , Rao, P. S. C. , Basu, N. B. , & Wilson, J. S. (2010). The future of hydrology: An evolving science for a changing world. Water Resources Research, 46, W05301 10.1029/2009WR008906

[wrcr24091-bib-0249] Walker, B. , Holling, C. S. , Carpenter, S. R. , & Kinzig, A. (2004). Resilience, adaptability and transformability in social–ecological systems. Ecology and Society, 9(2). 10.5751/ES-00650-090205

[wrcr24091-bib-0250] Wang, R. , Zimmerman, J. B. , Wang, C. , Vivanco, D. F. , & Hertwich, E. G. (2017). Freshwater vulnerability beyond local water stress: Heterogeneous effects of water‐electricity nexus across the continental United States. Environmental Science & Technology, 51(17), 9899–9910. 10.1021/acs.est.7b01942 28745496

[wrcr24091-bib-0251] Ward, N. K. , Fitchett, L. , Hart, J. A. , Shu, L. , Stachelek, J. , Weng, W. , Zhang, Y. , Dugan, H. , Hetherington, A. , Boyle, K. , Carey, C. C. , Cobourn, K. M. , Hanson, P. C. , Kemanian, A. R. , Sorice, M. G. , & Weathers, K. C. (2018). Integrating fast and slow processes is essential for simulating human–freshwater interactions. Ambio. 10.1007/s13280-018-1136-6 PMC6722150 30569439

[wrcr24091-bib-0252] WEF Water Initiative (2011). Water security: The water‐food‐energy‐climate nexus, World Economic Forum Water Initiative, (p. 39). Washington: Island Press.

[wrcr24091-bib-0253] Wei, J. , Wei, Y. , & Western, A. (2017). Evolution of the societal value of water resources for economic development versus environmental sustainability in Australia from 1843 to 2011. Global Environmental Change, 42, 82–92. 10.1016/j.gloenvcha.2016.12.005

[wrcr24091-bib-0254] Werner, B. T. , & McNamara, D. E. (2007). Dynamics of coupled human‐landscape systems. Geomorphology, 91(3‐4), 393–407. 10.1016/j.geomorph.2007.04.020

[wrcr24091-bib-0255] Wesselink, A. , Kooy, M. , & Warner, J. (2016). Sociohydrology and hydrosocial analysis: Towards dialogues across disciplines. WIREs Water, 4, e1196.

[wrcr24091-bib-0256] Western, A. W. , Blöschl, G. , & Grayson, R. B. (1998). How well do indicator variograms capture the spatial connectivity of soil moisture? Hydrological Processes, 12(12), 1851–1868. 10.1002/(SICI)1099-1085(19981015)12:12<1851::AID-HYP670>3.0.CO;2-P

[wrcr24091-bib-0257] Weststrate, J. , Dijkstra, G. , Eshuis, J. , Gianoli, A. , & Rusca, M. (2018). The Sustainable Development Goal on water and sanitation: Learning from the Millennium Development Goals. Social Indicators Research, 143(2), 795–810.

[wrcr24091-bib-0258] White, G. F. (1945). Human Adjustments to Floods, Department of Geography Research Paper, (Vol. 29, p. 225). Chicago: Department of Geography, University of Chicago.

[wrcr24091-bib-0259] Wind, H. G. , Nierop, T. M. , de Blois, C. J. , & de Kok, J. L. (1999). Analysis of flood damages from the 1993 and 1995 Meuse floods. Water Resources Research, 35(11), 3459–3465. 10.1029/1999WR900192

[wrcr24091-bib-0260] Winiwarter, V. , Schmid, M. , Haberl, H. , & Singh, S. J. (2016). Why legacies matter: Merits of a long‐term perspective In Haberl H., et al. (Eds.), Social ecology. Human‐environment interactions, (Vol. 5, pp. 149–168). Cham: Springer.

[wrcr24091-bib-0261] Winkel, L. , Berg, M. , Amini, M. , Hug, S. J. , & Johnson, C. A. (2008). Predicting groundwater arsenic contamination in Southeast Asia from surface parameters. Nature Geoscience, 1(8), 536–542. 10.1038/ngeo254

[wrcr24091-bib-0262] World Bank (2018). Somalia drought impact & needs assessment. Retrieved on 5 April 2019 at https://reliefweb.int/sites/reliefweb.int/files/resources/122991‐v1‐GSURR‐Somalia‐DINA‐Report‐Volume‐I‐180116‐Digital.pdf

[wrcr24091-bib-0263] Yillia, P. T. (2016). Water‐energy‐food nexus: Framing the opportunities, challenges and synergies for implementing the SDGs. Österreichische Wasser‐und Abfallwirtschaft, 68(3‐4), 86–98. 10.1007/s00506-016-0297-4

[wrcr24091-bib-0264] Yoffe, S. , Wolf, A. T. , & Giordano, M. (2003). Conflict and cooperation over international freshwater resources: Indicators of basins at risk. Journal of the American Water Resources Association (JAWRA), 39(5), 1109–1126. 10.1111/j.1752-1688.2003.tb03696.x

[wrcr24091-bib-0265] Yu, D. J. , Qubbaj, M. R. , Muneepeerakul, R. , Anderies, J. M. , & Aggarwal, R. M. (2015). Effect of infrastructure design on commons dilemmas in social−ecological system dynamics. Proceedings of the National Academy of Sciences, 112(43), 13,207–13,212. 10.1073/pnas.1410688112 PMC4629374 26460043

[wrcr24091-bib-0266] Yu, D. J. , Sangwan, N. , Sung, K. , Chen, X. , & Merwade, V. (2017). Incorporating institutions and collective action into a sociohydrological model of flood resilience. Water Resources Research, 53, 1336–1353. 10.1002/2016WR019746

[wrcr24091-bib-0267] Yu, D. J. , Shin, H. C. , Pérez, I. , Anderies, J. M. , & Janssen, M. A. (2017). Learning for resilience‐based management: Generating hypotheses from a behavioral study. Global Environmental Change, 37, 69–78.

[wrcr24091-bib-0268] Zeitoun, M. , & Mirumachi, N. (2008). Transboundary water interaction I: Reconsidering conflict and cooperation. International Environmental Agreements: Politics, Law and Economics, 8(4), 297–316. 10.1007/s10784-008-9083-5

[wrcr24091-bib-0269] Zhang, Z. , Hu, H. , Tian, F. , Yao, X. , & Sivapalan, M. (2014). Groundwater dynamics under water‐saving irrigation and implications for sustainable water management in an oasis: Tarim River basin of western China. Hydrology and Earth System Sciences, 18(10), 3951–3967. 10.5194/hess-18-3951-2014

[wrcr24091-bib-0271] Zwarteveen, M. , Kemerink‐Seyoum, J. S. , Kooy, M. , Evers, J. , Guerrero, T. A. , Batubara, B. , Biza, A. , Boakye‐Ansah, A. , Faber, S. , Cabrera Flamini, A. , & Cuadrado‐Quesada, G. (2017). Engaging with the politics of water governance. Wiley Interdisciplinary Reviews: Water, 4(6), e1245 10.1002/wat2.1245

[wrcr24091-bib-0272] Zwarteveen, M. Z. , & Boelens, R. (2014). Defining, researching and struggling for water justice: Some conceptual building blocks for research and action. Water International, 39(2), 143–158. 10.1080/02508060.2014.891168

